# Differential gene expression in the calvarial and cortical bone of juvenile female mice

**DOI:** 10.3389/fendo.2023.1127536

**Published:** 2023-06-12

**Authors:** Jerome Nicolas Janssen, Rotem Kalev-Altman, Tali Shalit, Dalit Sela-Donenfeld, Efrat Monsonego-Ornan

**Affiliations:** ^1^ The Institute of Biochemistry, Food Science and Nutrition, The Faculty of Agriculture, Food and Environment, The Hebrew University of Jerusalem, Rehovot, Israel; ^2^ The Koret School of Veterinary Medicine, The Faculty of Agriculture, Food and Environment, The Hebrew University of Jerusalem, Rehovot, Israel; ^3^ The Ilana and Pascal Mantoux Institute for Bioinformatics, The Nancy and Stephen Grand Israel National Center for Personalized Medicine, Weizmann Institute of Science, Rehovot, Israel

**Keywords:** juvenile, development, mouse, cortical, calvarial, transcriptome, locomotion

## Abstract

**Introduction:**

Both the calvarial and the cortical bones develop through intramembranous ossification, yet they have very different structures and functions. The calvaria enables the rapid while protected growth of the brain, whereas the cortical bone takes part in locomotion. Both types of bones undergo extensive modeling during embryonic and post-natal growth, while bone remodeling is the most dominant process in adults. Their shared formation mechanism and their highly distinct functions raise the fundamental question of how similar or diverse the molecular pathways that act in each bone type are.

**Methods:**

To answer this question, we aimed to compare the transcriptomes of calvaria and cortices from 21-day old mice by bulk RNA-Seq analysis.

**Results:**

The results revealed clear differences in expression levels of genes related to bone pathologies, craniosynostosis, mechanical loading and bone-relevant signaling pathways like WNT and IHH, emphasizing the functional differences between these bones. We further discussed the less expected candidate genes and gene sets in the context of bone. Finally, we compared differences between juvenile and mature bone, highlighting commonalities and dissimilarities of gene expression between calvaria and cortices during post-natal bone growth and adult bone remodeling.

**Discussion:**

Altogether, this study revealed significant differences between the transcriptome of calvaria and cortical bones in juvenile female mice, highlighting the most important pathway mediators for the development and function of two different bone types that originate both through intramembranous ossification.

## Introduction

The calvaria, the upper structure of the skull, allows fast and protected brain growth without heavy load bearing. Conversely, the cortical bone, which is the external layer of long bones, is crucial for locomotion and is constantly remodeled under the influence of weight loading. Despite differences in structure and function, both bones develop through the mechanism of intramembranous ossification, which initiates in the embryo and extensively progresses in neonates (1). The shared mode of formation of the cortical and calvarial bone, coupled with their distinct structures and functions, raises the fundamental question of how similar or diverged the molecular pathways that control their post-natal growth are. Investigating these mechanisms will enable a better understanding of the fundamental connection between shape and function and unravel the origin of specific bone-type diseases.

The process of intramembranous ossification is initiated by the condensation of mesenchymal cells in the flat bones of the skull and in the cortices of long bones. Following the differentiation of these mesenchymal cells into osteoblast (2, 3), a collagen-proteoglycan matrix becomes deposited, which enables the binding of calcium salts for the calcification of the bone matrix. Once embedded into the calcified bone matrix, osteoblasts differentiate into osteocytes regulating the mass and shape of the various types of bone (1).

In mice, the calvaria is formed by several skull bones connected through cranial sutures derived from the cranial neural crest and head mesoderm (4). Calvarial growth is the most intense during the first 9 post-natal days, but parameters like calvaria width still increase up to 25 post-natal days (5). The time for the fusion of the posterior frontal suture has been reported to end between 13 and 45 post-natal days (6, 7). However, sutures like the lambdoid suture remain unfused throughout life in mice (8).

On the other hand, the femoral cortical bone originates from the porous bone collar formed by osteoblasts, which derives from the lateral plate mesoderm during the early stages of bone development (9). In C57BL/6J mice, the cortical area increases nonlinearly between birth and 112 post-natal days. In contrast, the cortical thickness increases linearly from 14 to 112 post-natal days, indicating the switch from primarily bone formation to bone remodeling (10). Furthermore, the cortical bone grows by forming alternating struts and woven bone rings before becoming remodeled into a dense lamellar structure after birth (11). It matures during the process of secondary mineralization characterized by collagen compaction and changes in osteocyte gene expression (12).

Despite the shared process of intramembranous bone formation at early stages, differences between the extra-cellular matrix (ECM) of long bones and calvaria were observed in mature mice. For example, collagen was found to be more abundant in the calvaria and suggested to be less crosslinked. Contrary to this, the collagenous matrix of the long bones is more mineralized, probably providing an increased strength to the long bone that is necessary for weight bearing (13).

The growth and homeostasis of the cortical and calvarial bones are regulated by various signaling pathways, hence mutations in several signaling pathway mediators lead to distinct skeletal diseases that are shared between human and mice, making the latter a useful model for research (14). Other gene mutations or diseases are known to only affect one type of bone but not the other. For instance, the prevalent disease osteoporosis affects intensively the cortical bone, while the calvaria bone appear to be osteoporosis resistant (15). Yet, these studies focused on specific genes and diseases, hence systematic comparative studies are needed to fully uncover the shared or distinct molecular pathways in the two bone types at different stages.

Previous studies on adult mice, rats and macaques have compared gene expression between cortical and calvaria bones (16, 17). Both studies reported differential expression of genes related to body patterning and the WNT signaling pathway. However, these studies were done in mature bones undergoing remodeling rather than during their development and modeling. Moreover, both studies were performed on bone after the removal of periosteum and sutures, despite the suggested involvement of these tissues in intramembranous ossification (18). Hence, comprehensive knowledge regarding differential gene expression during the development and modeling of calvarial and cortical bones is missing.

This study focused on the post-natal developmental period by investigating juvenile cortical and calvarial bones. We used bulk RNA-Sequencing to analyze the transcriptome of 7 calvaria and 7 femoral cortical samples of 21 days old juvenile female mice to identify commonalities and differences in the expression level of genes associated with bone development, disease, weight loading and homeostasis. We further highlight differences between the juvenile and mature bone as well as differences in bone relevant signaling pathways observed between the juvenile murine calvaria and cortices.

## Material and methods

### Animals and RNA-sequencing

As described in Kalev-Altman et al. (19), female wild-type (WT) C57BL/6J (RRID: IMSR_JAX:000664) mice were purchased from Jackson laboratories (Rehovot, Israel). Mice were maintained at the Hebrew University Specific Pathogen Free animal facility according to animal care regulations. All procedures were approved by the Hebrew University Animal Care Committee (license number 21-16657-3). For growth analyses (n>10 for each weight measurement and n=7-12 for other measurements), animals were weighed weekly from P0 to 4 weeks of age and then biweekly. Body length was measured from nose to end of the sacral bone at P0 using the µCT software (Bruker, Kontich, Belgium) or from nose to tail at all other groups of age using a standard office ruler. Their skull and femur were dissected and cleaned from adjacent tissues. The femur length as well as all measurements of skull bones and long bones of P0 animals were all measured using the Amira software 3D measuring tool.

For RNA-Sequencing, mice were euthanized with CO2 at three weeks (P21). Cortical samples (n=7) were cleaned from all adjacent tissues (muscle, tendon and ligaments) and the diaphyseal bone marrow was removed by washing it out with ultra-pure water, using a small needle and syringe. Periosteum and endosteum were not removed. The left or right frontal bone was taken for the calvaria samples (n=7) without removing the periosteum or adjacent suture mesenchyme. The samples were individually flash-frozen in liquid nitrogen and were subjected to manual pulverization. Total RNA was extracted as previously described (20, 21). Each freshly frozen RNA sample had a RIN>5.6. Libraries were prepared by the Nancy and Stephen Grand Israel National Center for Personalized Medicine (G-INCPM) research facility, Weizmann Institute of Science, Rehovot, Israel; using INCPM-mRNA-seq, which is based on the Transeq protocol (22, 23). Sequencing was done on an Illumina NovaSeqmachine, using SP (100 cycles) protocol. The output was ~9.5 million single-end 100bp reads per sample. The RNA-Seq datasets are available at NCBI Gene Expression Omnibus (GEO), GEO accession: GSE223750.

### Bioinformatical analysis

Bioinformatical analysis was performed as previously described (20) with the following modifications: The EndToEnd option was used and outFilterMismatchNoverLmax was set to 0.05. Expression levels for each gene were quantified using htseq-count (version 0.11.2) (24) and using the gene annotations downloaded from Ensemble (release 102). Non-annotated genes were excluded. Differentially expressed genes were determined by a p.adj of <0.05, absolute fold changes >=1.5 and max counts >=15. PCA and PERMANOVA were performed based on the 1000 most variable genes, using R 4.2.0 and the vegan package (2.6-2). GOrilla website application (25) was used with default parameters using the list of DEGs and all genes detected having max counts >=15 as the background list. Gene set enrichment analysis (GSEA) was conducted using R packages clusterProfiler (3.18.1) and Org.Mm.eg.db (3.12.0) with FDR correction. Figures were created using DOSE (3.16.0) and PathView (1.30.1) (26).

## Results

### Gene set introduction

**Figure 1:**
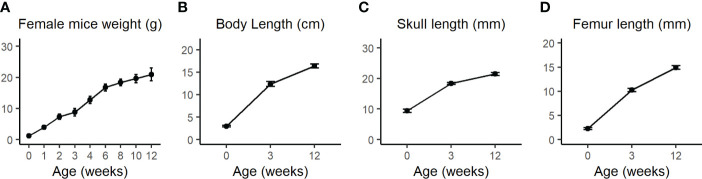
**(A)** Body weight of WT female mice was measured once a week from birth until 4 weeks (w) and then every two weeks until the age of 12w. **(B)** Total body length from nose to the end of the sacral bone at P0 as measured by the Amira software or from nose to tail at 3w and 12w as measured by a standard office ruler. **(C)** Total skull length measured by the Amira software 3D measuring tool. **(D)** Femur length was measured by the CTAn software. Values are expressed as the mean ± SD; n>10 for each weight measurement and n=7-12 for other measurements. Modified from Kalev-Altman et al. (19).

The calvarial and femoral cortical bone transcriptome data subset of 21 days old female C57BL/6J WT mice from Kalev-Altman et al. (19) was used and re-analyzed. This age is equivalent to ~6 months for humans, considering the average weaning period for mice and humans (27). **Figure 1** shows the assessment of weight, body length, skull length and femur length from P0 to 3M, demonstrating that calvaria and cortices of 21 days old mice are still subject to major bone growth and modeling (**Figures 1A–D**, respectively).

**Figure 2:**
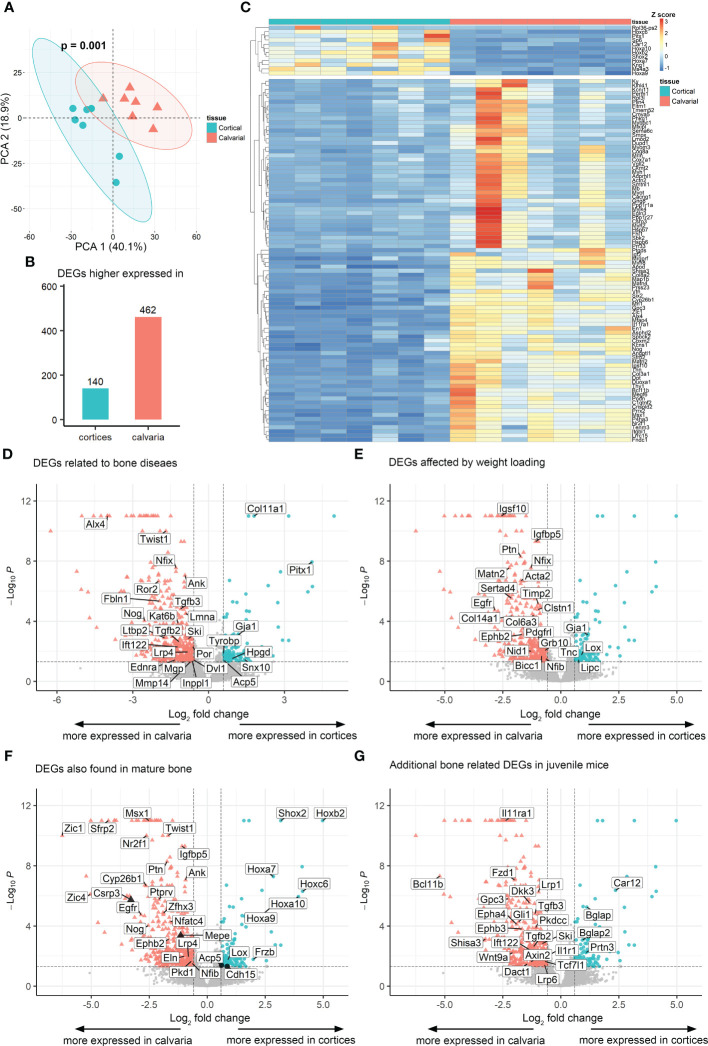
Transcriptome of cortical and calvarial bone of juvenile mice. **(A)** PCA analysis and PERMANOVA on centroids demonstrated that samples clustered according to tissue. 95% confidence ellipses of centroids are indicated. **(B)** Heatmap displaying the Z score of the 100 most variable differentially expressed genes (DEGs) between cortical and calvarial bone clustered according to the tissue of origin. **(C)** Total number of DEGs found to be higher expressed in either the cortices or the calvaria. **(D–G)** Volcano plots indicate the distribution of DEGs in cortical and calvarial bone. Annotated DEGs related to **(D)** diseases (14), **(E)** weight loading (28) and **(F)** DEGs that were also found to be differentially expressed between mature calvaria and cortices by Rawlinson et al. (17) or Wang et al. (16). DEGs with inverted expression levels compared to Rawlinson et al. (17) are colored in black. **(G)** Additional bone–associated DEGs in juvenile mice that are not differentially expressed in mature animals. DEGs were defined by total fold change >= 1.5, p.adj < 0.05 and maximal transcript number in at least one sample >=15.

PCA analysis of the 1000 most variable genes revealed a clustering of the calvarial and cortical bone samples, without overlapping samples from the different tissues (**Figure 2A**). PERMANOVA on the clusters’ centroids showed a significant difference between the samples from cortices and calvaria. The list of the top 100 expressed genes by transcript number for both tissues is shown in TabS1. As expected, bone-related genes such as *Collagen type I alpha 1* (*Col1a1*), *Col1a2*, *Serpin family F member 1* (*Serpinf1*), *Bone gamma-carboxyglutamate protein 1* (*Bglap1*), *Bglap2*, *Prolyl 4-hydroxylase subunit beta* (*P4hb*), *Osteonectin* (*Sparc*) (13) were detected in both bones. Furthermore, the expression of genes related to bone development and osteochondrogenic signaling pathways, such as bone morphogenetic protein (BMP), wingless-related integration site (WNT) and hedgehog (HH) (29) is shown in **Table S2** and provides an overview of the bone relevant pathways present in each type of bone.

Moreover, from the 10569 genes found, 602 differentially expressed genes (DEGs) were detected between the calvaria and the cortices. Of these 140 DEGs were higher expressed and 462 were lower expressed in the cortical bone compared to the calvaria (**Figure 2B**; **Table S3**). The expression of the top 100 DEGs by log_2_ fold change indicated two clusters of genes (**Figure 2C**). The upper cluster shows higher expression of DEGs in the cortical bone including several members of the *Homeobox* (*Hox*) family of transcription factors, while in the bottom cluster DEGs, such as the BMP inhibitor *Noggin* (*Nog*), were higher expressed in the calvaria.

### Differential expression of disease related genes in cortices and calvaria

As the first step of identifying DEGs that are highly relevant for bone formation, we compared our list of DEGs with the review of Brommage and Ohlsson (14), which summarized 441 genes related to human skeletal diseases including aggrecanopathies, channelopathies, ciliopathies, cohesinopathies, laminopathies, linkeropathies, lysosomal storage diseases, protein-folding and RNA splicing defects and ribosomopathies. Despite the lack of studies on several of these genes in mice, their subsequent literature search found 249 genes mimicking the human phenotype in mice. The expression of these conserved genes must be tightly regulated to prevent the occurrence of skeletal diseases. Therefore, a difference in their gene expression level between the cortical and calvarial bones may indicate an important role in site-specific bone development. We found 28 disease related DEGs including patterning and differentiation related transcriptions factors such as Paired-like homeodomain transcription factor 1 (Pitx1) and Twist basic helix-loop-helix transcription factor 1 (Twist1), the epigenetic regulator RUNX2 interacting histone lysine acetyltransferase 6B (Kat6b), general constituents of the bone matrix including Matrix gla protein (Mgp) and Col11a1, the osteoclast marker Acid phosphatase 5 tartrate resistant (Acp5), the WNT signaling associated Lipoprotein receptor-related protein 4 (Lrp4) and the ECM remodeling associated Matrix metallopeptidase (Mmp14) (14) (**Figure 2D**; **Table 1**). The difference in expression levels of disease related genes associated with bone cell differentiation, bone matrix composition and remodeling between calvarial and cortical bone are likely relevant for the bones’ diverging function, e.g., resisting heavy loading of the cortices for locomotion (30) or providing a permanent and robust protection of the brain by the calvarial bone matrix.

**Table 1:** Bone pathology associated DEGs as reported by Brommage and Ohlsson (14).

Gene ID	Gene name	log2FC (Cortical/Calvarial)	p.adj
ENSMUSG00000021506	Pitx1	4.09	1.15E-08
ENSMUSG00000027966	Col11a1	1.80	0.00E+00
ENSMUSG00000038301	Snx10	1.42	3.54E-02
ENSMUSG00000050953	Gja1	1.17	8.40E-04
ENSMUSG00000031613	Hpgd	0.93	3.10E-02
ENSMUSG00000001348	Acp5	0.62	4.09E-02
ENSMUSG00000030579	Tyrobp	0.59	1.35E-02
ENSMUSG00000029071	Dvl1	-0.59	3.76E-02
ENSMUSG00000005514	Por	-0.63	5.07E-03
ENSMUSG00000032737	Inppl1	-0.65	4.32E-02
ENSMUSG00000000957	Mmp14	-0.71	4.91E-02
ENSMUSG00000029050	Ski	-0.81	1.49E-03
ENSMUSG00000027253	Lrp4	-0.82	1.12E-02
ENSMUSG00000028063	Lmna	-0.91	3.03E-05
ENSMUSG00000022265	Ank	-0.94	8.85E-08
ENSMUSG00000030218	Mgp	-0.97	4.89E-02
ENSMUSG00000021253	Tgfb3	-1.11	1.54E-05
ENSMUSG00000039239	Tgfb2	-1.17	2.38E-03
ENSMUSG00000021767	Kat6b	-1.21	1.39E-04
ENSMUSG00000030323	Ift122	-1.21	5.20E-03
ENSMUSG00000001911	Nfix	-1.29	2.89E-08
ENSMUSG00000031616	Ednra	-1.32	3.76E-02
ENSMUSG00000035799	Twist1	-1.69	1.00E-10
ENSMUSG00000002020	Ltbp2	-1.74	1.31E-03
ENSMUSG00000006369	Fbln1	-1.94	4.56E-06
ENSMUSG00000021464	Ror2	-1.98	2.18E-07
ENSMUSG00000048616	Nog	-2.54	9.25E-05
ENSMUSG00000040310	Alx4	-4.01	0.00E+00

### Mechanical loading-associated genes are upregulated in the calvaria

Mechanical loading is a major effector in the modeling and remodeling of bones (30). Thus, we expected significant differences in the expression of mechanical load-associated genes between the load-bearing cortices and non-load-bearing calvaria. Accordingly, we compared our list of DEGs to the list of loading-associated genes reported by Xing et al. (28), which studied the tibia of 10-week-old female mice. 22 of our DEGs were associated with mechanical loading. Four genes were higher expressed in the cortical bone. These include the Lysyl oxidase (Lox), whose crosslinking activity has been proposed to give collagen fibers more stiffness that may be necessary for locomotion (13) and the WNT-related Gap junction protein alpha 1 (Gja1). However, the majority of 18 genes were higher expressed in the calvaria, including the Growth factor receptor bound protein 10 (Grb10), other genes involved in ECM cell signaling as reported for Nidogen 1 (Nid1), ECM constituents like Col14a1 and the tissue inhibitor of metalloproteinase 2 (Timp2) that could play a role in preventing matrix degradation due to a lack of weight loading in the skull (**Figure 2E**; **Table 2**).

**Table 2:** DEGs associated with mechanical loading as reported by Xing et al. (28).

Gene ID	Gene name	log2FC (Cortical/Calvarial)	p.adj
ENSMUSG00000050953	Gja1	1.17	8.40E-04
ENSMUSG00000024529	Lox	0.89	1.79E-02
ENSMUSG00000032207	Lipc	0.76	3.60E-02
ENSMUSG00000028364	Tnc	0.70	2.55E-02
ENSMUSG00000008575	Nfib	-0.65	3.06E-02
ENSMUSG00000020176	Grb10	-0.68	9.01E-03
ENSMUSG00000014329	Bicc1	-0.83	2.01E-02
ENSMUSG00000031595	Pdgfrl	-0.91	2.29E-03
ENSMUSG00000017466	Timp2	-0.98	7.23E-06
ENSMUSG00000039953	Clstn1	-0.99	1.69E-05
ENSMUSG00000026185	Igfbp5	-1.09	5.00E-10
ENSMUSG00000048126	Col6a3	-1.17	4.06E-05
ENSMUSG00000005397	Nid1	-1.27	1.02E-02
ENSMUSG00000001911	Nfix	-1.29	2.89E-08
ENSMUSG00000028664	Ephb2	-1.66	7.67E-04
ENSMUSG00000029838	Ptn	-1.73	5.20E-09
ENSMUSG00000035783	Acta2	-1.73	2.07E-07
ENSMUSG00000022371	Col14a1	-2.10	4.42E-05
ENSMUSG00000016262	Sertad4	-2.13	2.96E-06
ENSMUSG00000022324	Matn2	-2.22	2.82E-08
ENSMUSG00000036334	Igsf10	-2.57	0.00E+00
ENSMUSG00000020122	Egfr	-2.85	1.85E-05

### Commonalities and differences between juvenile and mature calvaria and cortices

Juvenile and adolescent bone differ in various processes, such as bone elongation. Hence, we sought to investigate if the DEGs found between the juvenile calvaria and cortices overlap with DEGs found between adult calvaria and cortices. We compared our list of DEGs obtained from 21 days old female mice to the combined 225 DEGs found by Wang et al. (16), who analyzed calvaria and tibia osteocytes in 4-month old mice, 6-month old rats and 6-year old macaque using RNA-Seq and Rawlinson et al. (17), who compared skull and limb bones of rats as well as of mature mice by microarray. Our study included bones with adjacent suture tissue, periosteum and endosteum, as these tissues are involved in intramembranous ossification (18). It should be considered that both studies on mature animals removed suture tissue and periosteum, therefore impeding the comparison with these studies. From 225 DEGs reported in their studies, 135 genes were also detected in our RNA-Seq.

First, we found several DEGs with a higher expression level in the calvaria as compared to the cortical bone in juvenile animals, which was also reported for mature animals. These include, among others, Insulin-like growth factor binding protein 5 (Igfbp5), Lrp4, Msh homeobox 1 (Msx1) and Nog (**Figure 2F**; **Table S4**). Additionally, Rawlinson et al. (17), found a significantly higher expression level of several DEGs in the skull compared to the limb, such as Lrp5 and Sclerostin (Sost). However, we observed only a non-significant elevated expression of these genes in our study, which may also result from differences in study design that will be elaborated on in the discussion.

Next, to further address commonalities between adolescent and mature bone, we focused on genes with a higher expression level in the cortical bone as compared to the calvaria. In agreement with the studies mentioned above on mature bone, we found DEGs such as Frizzled-related protein (Frzb), Short stature homeobox 2 (Shox2) and several HOX genes having a significantly higher expression level in the juvenile cortical bone compared to the calvaria. Furthermore, our analysis of juvenile bone revealed only a non-significant higher expression level of genes such as Cartilage oligomeric matrix protein (Comp) and Catenin beta 1 (Ctnnb1) in the cortical bone compared to the calvaria. Their expression levels were found to be significantly higher in Wang et al. (16) and Rawlinson et al. (17) studies on mature animals contrary to our study on juvenile mice.

At variance with the expression levels observed by Rawlinson et al. (17) investigating mature bone, we detected higher expression levels of the osteogenic marker Matrix extra-cellular phosphoglycoprotein (Mepe) and cardiomyopathy related Cysteine and glycine-rich protein 3 (Csrp3) in the calvaria compared to the cortical bone. Hitherto, no reports on Csrp3 function in bone cells exist, but as seen for their role in the heart, a function in mechanical sensing is possible (31). Additionally, we detected lower expression levels of the osteoclast marker Acp5 and skeletal muscle marker Cadherin 15 (Cdh15) in the juvenile calvarial bone compared to the cortical bone. It was previously shown that C2C12 myoblasts which undergo osteogenic differentiation following BMP2 treatment suppress CDH15 protein level (32). However, it should be considered that our whole tissue RNA-Seq approach may not be sensitive enough to detect differences in low abundant cell populations like osteoclasts. Single cell RNA-Seq may overcome this limitation, although it has recently been described as not yet sensitive enough for low abundant bone cells (33). In total, several DEGs between cortical and calvarial bone exhibited the same expression pattern in juvenile and in mature bone, while only 4 DEGs between the cortical and calvarial bone had opposing expression levels comparing juvenile and mature bone.

Moreover, we detected additional bone or ECM related DEGs that were not detected or categorized as being differentially expressed by Wang et al. (16) and Rawlinson et al. (17). Some of these DEGs may be related to the non-removal of the periosteum and sutures. Amongst others Bglap, Bglap2 (13), Carbonic anhydrase 12 (Car12) (34) and Proteinase 3 (Prtn3) (35) exhibited a higher expression level in the cortical bone compared to the calvaria (**Figure 2G**; **Table 3**). Moreover, the calvaria demonstrated higher expression levels compared to the cortical bone of several mediators linked to the BMP, WNT and HH signaling pathways that have been associated with bone development (29). These differences in expression level of their mediators may play an important role in fine-tuning bone growth according to their function. For example, Transforming growth factor beta 2 (Tgfb2) and Tgfb3 are both linked to skeletal diseases (14). Next, the WNT-associated Shisa3 has been reported by Murakami et al. (44) to be expressed in calvarial osteoblasts, although no phenotype was reported after Shisa3 knockout. Dickkopf 3 (Dkk3) has been previously proposed to inhibit endochondral bone formation (42), but as with WNT related Transcription factor 7 like 1 (Tcf7l1) (39) and Dishevelled-binding antagonist of beta-catenin 1 (Dact1) (43), no study investigated its role in calvaria or cortical bone development in juvenile mice. The HH pathway mediators GLI family zinc finger 1 (Gli1) that promotes osteoblast differentiation (48), Intraflagellar transport 122 (Ift122) that was linked to cranioectodermal dysplasia in humans and protein kinase domain containing cytoplasmic (Pkdcc), which regulates craniofacial and long bone development (46) demonstrated higher expression levels in the calvaria compared to cortices. Finally, additional genes associated with craniosynostosis were strongly expressed in the calvaria compared to the cortical bone, such as Axin2 (6), B cell leukemia/lymphoma 11B (Bcl11b) (49), Interleukin 11 receptor alpha chain 1 (Il11ra1) and Ski sarcoma viral oncogene homolog (Ski) (4), as well as the ephrin receptors Epha4 and Ephb3.

**Table 3:** Additional bone related DEGs found comparing juvenile murine cortices and calvaria.

Category	Gene ID	Gene name	log2FoldChange (Cortical/Calvarial)	p.adj	References
Uncategorized	ENSMUSG00000032373	Car12	2.36	4.30E-07	Liu et al. (34)
	ENSMUSG00000074483	Bglap	1.12	5.58E-06	van den Bos et al. (13)
	ENSMUSG00000057729	Prtn3	1.09	5.95E-03	Shao et al. (35)
	ENSMUSG00000074486	Bglap2	0.93	8.84E-04	van den Bos et al. (13)
	ENSMUSG00000026072	Il1r1	-0.85	1.67E-02	Matsuda et al., 2010 (36)
BMP signaling	ENSMUSG00000021253	Tgfb3	-1.11	1.54E-05	Guasto and Cormier-Daire, (29)
	ENSMUSG00000039239	Tgfb2	-1.17	2.38E-03	Guasto and Cormier-Daire, (29)
	ENSMUSG00000055653	Gpc3	-2.26	1.52E-06	Dwivedi et al. (37); Kolluri and Ho, 2019 (38)
WNT signaling	ENSMUSG00000055799	Tcf7l1	-0.69	2.27E-02	Velasco et al. (39)
	ENSMUSG00000030201	Lrp6	-0.70	3.82E-02	Maupin et al. (40)
	ENSMUSG00000040249	Lrp1	-0.96	5.54E-07	Lu et al. (41)
	ENSMUSG00000030772	Dkk3	-1.35	2.96E-06	Aslan et al., 2006, (42); Maupin et al. (40)
	ENSMUSG00000044548	Dact1	-1.44	3.31E-02	Esposito et al. (43)
	ENSMUSG00000044674	Fzd1	-1.99	8.85E-08	Maupin et al. (40)
	ENSMUSG00000000126	Wnt9a	-2.08	5.92E-03	Maupin et al. (40)
	ENSMUSG00000050010	Shisa3	-3.25	7.54E-04	Murakami et al. (44)
HH signaling	ENSMUSG00000032855	Pkd1	-0.69	2.22E-02	Qiu et al. (45)
	ENSMUSG00000024247	Pkdcc	-1.08	1.30E-04	Maridas et al. (46)
	ENSMUSG00000030323	Ift122	-1.21	5.20E-03	Moosa et al., 2016 (47)
	ENSMUSG00000025407	Gli1	-1.82	5.33E-05	Ohba (48)
Craniosynostosis	ENSMUSG00000000142	Axin2	-1.29	2.47E-02	Behr et al. (6)
	ENSMUSG00000005958	Ephb3	-1.67	1.57E-04	Ishii et al. (4)
	ENSMUSG00000026235	Epha4	-1.90	7.72E-05	Ishii et al. (4)
	ENSMUSG00000073889	Il11ra1	-2.39	0.00E+00	Ishii et al. (4)
	ENSMUSG00000048251	Bcl11b	-5.22	4.91E-08	Holmes et al. (49)

In summary, we found over 600 DEGs between calvaria and cortical bone of femur in juvenile female mice, including DEGs that were detected in previous studies that focused on adult animals and differed in experimental procedures. Many of our bone related DEGs, like several WNT signaling mediators, were not categorized as DEGs in these studies, and many DEGs reported in these studies were not confirmed by us in juvenile animals.

### Enrichment of bone-relevant gene ontology terms in juvenile murine bone

Next, to check for enriched gene ontology (GO) Biological Process terms, we used GOrilla on the DEGs detected in this study. The resulting top 20 terms ranked by the enrichment score are shown in **Table 4**, including bone relevant terms related to protein kinase C activity (50), response to Vitamin K (51), forelimb morphogenesis, cartilage condensation and WNT signaling (52) (**Table 4**). DEGs that were associated with these gene sets include Epidermal growth factor receptor (Egfr), Receptor tyrosine kinase like orphan receptor 2 (Ror2), Bglap2, Twist1, Alx4, Wnt9a, Msx1, Lrp6, Ift122, Polycystin 1 (Pkd1) and Tgfb2, which exhibited a higher expression level in the calvaria compared to the cortical bone as well as Shox2, Hoxa9 and Col11a1, which exhibited a higher expression level in the cortical bone compared to the calvaria (**Figures 2D–G**). Our results indicate that multiple gene sets are responsible for the differential development of the juvenile murine calvarial and cortical bone.

**Table 4:** Enriched terms by Gorilla.

GO Biological Process Term	Description	p.value	FDR q-value	Enrichment Score	Genes
GO:1900020	positive regulation of protein kinase C activity	2.70E-04	2.25E-02	15.44	Agt - angiotensinogen (serpin peptidase inhibitor, clade a, member 8), Egfr - epidermal growth factor receptor, Ror2 - receptor tyrosine kinase-like orphan receptor 2
GO:1900019	regulation of protein kinase C activity	2.70E-04	2.24E-02	15.44	Agt - angiotensinogen (serpin peptidase inhibitor, clade a, member 8), Egfr - epidermal growth factor receptor, Ror2 - receptor tyrosine kinase-like orphan receptor 2
GO:1990048	anterograde neuronal dense core vesicle transport	2.70E-04	2.23E-02	15.44	Kif1c - kinesin family member 1c, Kif1b - kinesin family member 1b, Sybu - syntabulin (syntaxin-interacting)
GO:0060371	regulation of atrial cardiac muscle cell membrane depolarization	2.70E-04	2.21E-02	15.44	Cacna1g - calcium channel, voltage-dependent, t type, alpha 1g subunit, Scn1b - sodium channel, voltage-gated, type i, beta, Gja1 - gap junction protein, alpha 1
GO:0032571	response to vitamin K	8.27E-05	8.50E-03	12.35	Gas6 - growth arrest specific 6, Bglap - bone gamma carboxyglutamate protein, Bglap2 - bone gamma-carboxyglutamate protein 2, Postn - periostin, osteoblast specific factor
GO:0014820	tonic smooth muscle contraction	8.27E-05	8.44E-03	12.35	Mylk - myosin, light polypeptide kinase, Ednra - endothelin receptor type a, Cacna1g - calcium channel, voltage-dependent, t type, alpha 1g subunit, Agt - angiotensinogen (serpin peptidase inhibitor, clade a, member 8)
GO:0062042	regulation of cardiac epithelial to mesenchymal transition	5.21E-04	3.76E-02	8.82	Twist1 - twist basic helix-loop-helix transcription factor 1, Tgfb2 - transforming growth factor, beta 2, Nog - noggin, Eng - endoglin
GO:0014829	vascular smooth muscle contraction	5.21E-04	3.74E-02	8.82	Ednra - endothelin receptor type a, Acta2 - actin, alpha 2, smooth muscle, aorta, Cacna1g - calcium channel, voltage-dependent, t type, alpha 1g subunit, Agt - angiotensinogen (serpin peptidase inhibitor, clade a, member 8)
GO:0003071	renal system process involved in regulation of systemic arterial blood pressure	5.21E-04	3.72E-02	8.82	Gas6 - growth arrest specific 6, Emp2 - epithelial membrane protein 2, Agt - angiotensinogen (serpin peptidase inhibitor, clade a, member 8), Gja1 - gap junction protein, alpha 1
GO:0060312	regulation of blood vessel remodeling	5.21E-04	3.70E-02	8.82	Tmbim1 - transmembrane bax inhibitor motif containing 1, Flt4 - fms-like tyrosine kinase 4, Erg - avian erythroblastosis virus e-26 (v-ets) oncogene related, Gja1 - gap junction protein, alpha 1
GO:0035115	embryonic forelimb morphogenesis	2.87E-07	9.06E-05	8.17	En1 - engrailed 1, Msx1 - msh homeobox 1, Alx4 - aristaless-like homeobox 4, Wnt9a - wingless-type mmtv integration site 9a, Twist1 - twist basic helix-loop-helix transcription factor 1, Shox2 - short stature homeobox 2, Hoxa9 - homeobox a9, Lrp6 - low density lipoprotein receptor-related protein 6, Ift122 - intraflagellar transport 122
GO:0048251	elastic fiber assembly	2.15E-04	1.86E-02	7.72	Fbln5 - fibulin 5, Ltbp4 - latent transforming growth factor beta binding protein 4, Mfap4 - microfibrillar-associated protein 4, Lox - lysyl oxidase, Thsd4 - thrombospondin, type i, domain containing 4
GO:0033622	integrin activation	9.88E-04	5.99E-02	7.72	Cx3cl1 - chemokine (c-x3-c motif) ligand 1, Cxcl12 - chemokine (c-x-c motif) ligand 12, Fermt2 - fermitin family homolog 2 (drosophila), Col16a1 - collagen, type xvi, alpha 1
GO:0060065	uterus development	9.88E-04	5.96E-02	7.72	Hoxa10 - homeobox a10, Hoxa9 - homeobox a9, Rbp4 - retinol binding protein 4, plasma, Tgfb2 - transforming growth factor, beta 2
GO:0098743	cell aggregation	8.34E-05	8.45E-03	7.13	Thra - thyroid hormone receptor alpha, Mpz - myelin protein zero, Pkd1 - polycystic kidney disease 1 homolog, Tgfb2 - transforming growth factor, beta 2, Ror2 - receptor tyrosine kinase-like orphan receptor 2, Col11a1 - collagen, type xi, alpha 1
GO:0033273	response to vitamin	8.34E-05	8.39E-03	7.13	Gas6 - growth arrest specific 6, Ada - adenosine deaminase, Bglap - bone gamma carboxyglutamate protein, Bglap2 - bone gamma-carboxyglutamate protein 2, Lrp6 - low density lipoprotein receptor-related protein 6, Postn - periostin, osteoblast specific factor
GO:2000095	regulation of Wnt signaling pathway, planar cell polarity pathway	3.73E-04	2.88E-02	7.02	Nkd1 - naked cuticle 1 homolog (drosophila), Gpc3 - glypican 3, Sfrp2 - secreted frizzled-related protein 2, Lrp6 - low density lipoprotein receptor-related protein 6, Dact1 - dapper homolog 1, antagonist of beta-catenin (xenopus)
GO:0035136	forelimb morphogenesis	1.66E-06	3.76E-04	6.95	En1 - engrailed 1, Msx1 - msh homeobox 1, Alx4 - aristaless-like homeobox 4, Wnt9a - wingless-type mmtv integration site 9a, Twist1 - twist basic helix-loop-helix transcription factor 1, Shox2 - short stature homeobox 2, Hoxa9 - homeobox a9, Lrp6 - low density lipoprotein receptor-related protein 6, Ift122 - intraflagellar transport 122
GO:0001502	cartilage condensation	6.06E-04	4.22E-02	6.43	Thra - thyroid hormone receptor alpha, Pkd1 - polycystic kidney disease 1 homolog, Tgfb2 - transforming growth factor, beta 2, Ror2 - receptor tyrosine kinase-like orphan receptor 2, Col11a1 - collagen, type xi, alpha 1
GO:0060004	reflex	6.06E-04	4.20E-02	6.43	Auts2 - autism susceptibility candidate 2, Npnt - nephronectin, Shank1 - sh3/ankyrin domain gene 1, Pmp22 - peripheral myelin protein 22, Gja1 - gap junction protein, alpha 1

### Enrichment of KEGG gene sets in the juvenile murine calvaria and cortices

**Figure 3:**
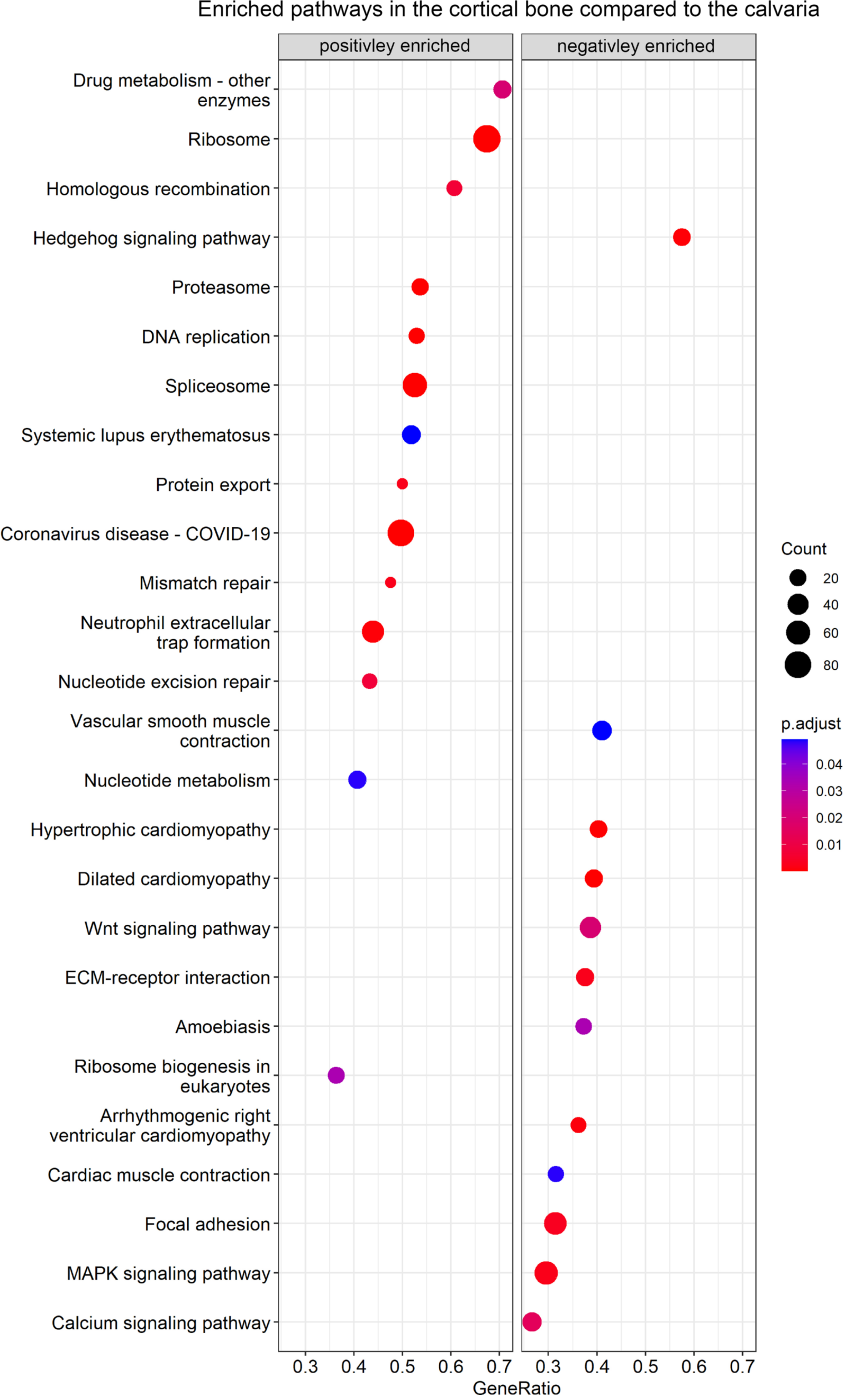
GSEA of enriched KEGG pathways between cortical and calvarial bone. The cortical bone displayed a positive enrichment for gene sets related to DNA replication, whereas the calvaria displayed a positive enrichment of gene sets related to signaling pathways. P–values were adjusted using FDR correction. Gene ratio is defined as the number of enriched genes in the gene set/total number of genes in the gene set and the enriched gene number per gene set is represented by count size.

In the next step, we performed GSEA using the KEGG database to identify enriched data sets by ranking all detected genes, including DEGs (**Figure 3**; **Table S5**). In total, 26 enriched gene sets were found, including 14 with a positive normalized enrichment score and 12 with a negative normalized enrichment score in the cortical bone compared to the calvaria.

Gene sets positively enriched in the cortical bone focused on cellular pathways and included nucleotide metabolism, homologous recombination, DNA replication, mismatch repair, spliceosome and ribosome-related terms. Moreover, the calvaria demonstrated a positive enrichment for terms focusing on signaling pathways such as calcium, Hedgehog, MAPK and WNT signaling as well as related terms like ECM-receptor interaction and Focal adhesion.

For instance, **Figure S1** shows the enriched gene sets of WNT/HH signaling, Ribosome and ECM-receptor interaction. Canonical and non-canonical WNT signaling pathways contained genes with higher and lower expression levels between the calvaria and cortices. Therefore no conclusion could be drawn as to which specific WNT pathway may be more activated in cortical bone or the calvaria (**Figure S1A**). Additionally, **Figure S1B** shows the increased expression of several HH mediators in the calvaria. The HH signaling gene set has been linked to craniofacial development and our list of DEGs contained the HH pathway mediators Gli1, Ift122, Pkd1 that effects Gli2 expression (45), and Pkdcc. All four genes were linked to the primary cilium (53–56), an organ that is involved in mechanosensing (57, 58), indicating another connection between the HH and WNT pathways regulating bone development (52). Furthermore, **Figures S1C, D** show the increased expression levels of ribosome-related genes in the cortical bone. These gene sets include the genes EMG1 N1-specific pseudouridine methyltransferase (Emg1), which is associated with the Bowen-Conradi syndrome (59), Treacle (Tcof1), which is associated with Treacher Collins syndrome as well as the Diamond-Blackfan anemia associated Ribosomal protein L11 (Rpl11), Rpl35a, Ribosomal protein S17 (Rps17) and Rps24 (60). Several integrins as well as ECM molecules like collagens and osteopontin were differentially expressed between cortices and calvaria (**Figure S1E**). For instance, we observed elevated Integrin alpha 5 (Itga5) and Integrin beta 1 (Itgb1) expression levels in the calvaria, and a decreased expression of these integrins was linked to apoptosis in unloaded rat osteoblasts (61). Furthermore, in agreement with the finding that αVβ3 integrins are highly abundant in osteoclasts (62), we observed an increased Itgav/Itgb3 integrins expression level in the cortical bone. Therefore, the enrichment of the ECM receptor gene set could indicate differences in osteoblast and osteoclast activity.

The MAPK signaling gene set was positively enriched in the calvaria and has been linked to osteogenesis (63) and WNT signaling (64). Although our list of DEGs did not contain the most abundant MAPK signaling mediators, it did include some distant mediators of the KEGG MAPK gene set such as Egfr, Interleukin 1 receptor type I (Il1r1), Tgfb2 and Tgfb3. Interestingly, MAPK is further related to the calvaria-enriched calcium signaling gene set. Moreover, Eapen et al. (65) reported that calcium signaling activates the p38 MAPK pathway, eventually inducing Runt-related transcription factor 2 (Runx2) expression and calvarial osteoblast differentiation *in vitro*. Finally, the calvaria showed a positive enrichment for gene sets related to cardiovascular-related terms compared to the cortices. This is not surprising as the enriched gene sets included integrins as well as DEGs like Lamin A/C (Lmna) (66) and Tenascin C (Tnc) (67), both taking part in bone formation as well as cardiovascular related gene sets.

## Discussion

In this study, we compared the transcriptome of two different bones, the skull calvaria and the femoral cortical bone. While both bones develop through intramembranous ossification, each is structurally and functionally different. The skull bone protects the brain, whereas the cortical bone is involved in locomotion and load-bearing. Moreover, each bone is affected by different diseases. Hence, the question regarding their similar or divergent molecular signature arises. Previous studies focused on embryological development (4, 11) and the mature state of these bones at various mammalian species (16, 17). Here, we used a high number of biological replicates to provide new insights into the juvenile murine bone, which still undergoes modeling processes. A limitation of this study was the analysis of only juvenile female mice. However, the sexual maturation for C57BL/6 mice has been reported to begin after P25 (68) and therefore, only minor differences to male mice can be expected. Notably, the bulk RNA-Seq was conducted on RNA extracted directly from bone tissue to remain as close as possible to the native tissue since isolation and cultivation of osteogenic cells *in-vitro* before RNA-Seq analysis was reported to affect gene expression levels (33). First, we scanned our list of DEG for genes that have been linked to bone disease, development and homeostasis in the literature. The GO analysis of all 602 DEGs showed expected results, e.g., biologically relevant terms related to limb development that match the higher expression levels of pattern-associated *Hox* genes in the cortices compared to the calvaria as reported for mature animals as well (16, 17). The detected DEGs are involved in various processes from epigenetic regulation up to matrix remodeling, rather than only one process, indicating that every step of bone modeling in calvaria and cortices can vary between both bones. Subsequently, we performed GSEA to identify enriched KEGG pathways in the cortical bone and calvaria.

### Several key bone markers are not differentially expressed between the calvaria and cortical bone of juvenile mice

Our study provided general oversight of the expression level of several genes related to bone development and homeostasis. We were not able to detect significant differences in the expression level of major bone genes between calvaria and cortical bones, such as Alkaline phosphatase (Alpl), Col1a1, Col1a2, Dentin matrix protein 1 (Dmp), Integrin binding sialoprotein (Ibsp), Runx1, Runx2, Runx3, Osterix (Sp7), Serpinf1 or Sparc. It seems likely that the expression levels of these universal bone markers are bone type independent and bone-specific differences occur in the subsequent processes, such as posttranscriptional regulation. At variance from the classical bone genes described above, many genes displayed significant differences in expression level between both bone types. For instance, the expression level of Lox was significantly higher in the cortical bone compared to the skull. This finding is in line with the increased cross-linked collagen in the cortical bone reported by van den Bos et al. (13). Furthermore, our observation of increased expression levels of the calcification associated genes Bglap, Bglap2 (13), Car12 (34) and Prtn3 (35) in the cortical bone compared to the calvaria supports the assumption that increased calcification occurs in locomotion-participating bones (13).

### High expression of synostosis-related genes in the calvaria

The calvaria originates, contrary to the cortical bone, from several bones bound together by cranial sutures to enable rapid while protected brain growth. Cranial sutures of mice remain mostly unfused. Our list of DEGs from juvenile mice contained several craniosynostosis-related genes whose expression was higher in the calvaria than in the cortical bone. The different sample preparation, including the removal of sutures as performed by Wang et al. (16) and Rawlinson et al. (17) on mature animals impedes the comparison of craniosynostosis relevant DEGs that were detected in their studies and the DEGs reported here, such as Axin2, Aristaless-like homeobox 4 (Alx4), Zic family member 1 (Zic1), the Glycoprotein glypican 3 (Gpc3) and ephrin receptors. Axin2 knockout lead to a reduction in hypertrophic chondrocytes and enhanced cranial suture mineralization and ossification, possibly by aberrant activation of WNT signaling (40). Alx4 interacts with BMPs as well as WNT-family proteins (69), whereas Zic1 is thought to induce engrailed (En) expression *via* WNT signaling (70). Gpc3 has been reported to modulate WNT and HH signaling in liver cancer (38) and interestingly, GPC3 was previously found to interact with BMP2, like the not differentially expressed GPC1, to regulate suture fusion. Yet, contrary to single knockouts, only a combined knockout of Gpc1 and Gpc3 led to a skull phenotype (37). This redundant function suggests that Gpc3 is only a minor regulator of bone development through fine-tuning of BMP signaling. Altogether, many craniosynostosis-related genes that are upregulated in the calvaria act *via* modulation of signaling pathways like the BMP and WNT signaling pathways. These pathways regulate osteogenic and chondrogenic differentiation events, which delay suture fusion until the growth of the brain is completed and ossification of the sutures, except for the lambdoid suture, takes place. The role of other craniosynostosis-related genes in the calvaria has not been completely understood yet. For example, Ephb3 has been reported to be present in the embryonic and adult calvaria sutures and an Ephb3 knockout increased calvaria bone tissue volume (71). Ephb2 was suggested to be involved in embryonic skull development; however, Ephb2 knockout mice did not show any calvaria phenotype (72). Therefore, Ephb2 may work together with Ephb3 as seen during palate formation (73) but downstream of Ephb3 during craniosynostosis. It has been suggested that adult calvarial sutures may act as reservoirs of Ephb3 osteoblastic stem cells responding upon brain injury. Thus, some detected DEGs might function in calvarial bone repair rather than calvarial bone formation.

### WNT signaling in the calvaria and the response to mechanical loading

Maupin et al. (40) reviewed the effects of mutations in WNT signaling genes on general bone development in human and mice, which included several of our DEGs, such as *Lrp4*, whose expression has been linked to increased bone mineral content and density. We found additional WNT signaling mediators with a significantly higher expression level in the calvaria, such as *Lrp1*, that is expressed in osteoblast regulating osteoclast activity (74). The osteoclast-specific loss of *Lrp1* increased osteoclastogenesis and bone resorption in mice (41). Therefore, the higher expression of *Lrp1* in the calvaria may have a pivotal role in preventing calvaria bone loss. The expression of WNT genes may not be limited to cells residing in the bone, as it was shown that periosteal stem cells express WNT genes during the growth phase (18), indicating an essential role of the periosteum in site-specific bone formation.

The WNT signaling pathway is not just crucial for bone development but also connects mechanical loading with bone remodeling. Surprisingly, most DEGs associated with mechanical loading had a higher expression level in the non-load-bearing calvaria than in the load-bearing cortical bone. In the cortical bone, mechanical loading leads to the expression of bone anabolic genes and prevents bone loss. This feedback mechanism cannot be applied to the non-load-bearing skull. Therefore, we suggest that at least a subset of these genes responsive to mechanical loading in the cortical bone is also involved in regulating bone homeostasis in the calvaria. This subset of genes including e.g., *Timp2* is constantly expressed by WNT signaling to prevent calvaria bone loss in juvenile mice. In addition, other load-inducible genes being highly expressed in the calvaria of mature animals, as demonstrated by Rawlinson et al. (17) may explain how the senescent skull does not suffer from osteoporosis (15).

We highlight the WNT pathway’s role in juvenile mice in osteogenic differentiation and subsequent bone mineralization during craniosynostosis, as well as WNT signaling as a response to mechanical loading and inhibiting bone loss in the calvaria. The gene expression data presented here identified WNT signaling as the most significant pathway to establish the differences in bone structure and function observed between the juvenile calvaria and cortical bone. While this study focused on differential expression of WNT pathway members and effectors, more research on the posttranscriptional level is necessary to fully comprehend how differences between the juvenile calvarial and cortical bone are established.

### DNA handling and ribosomal composition may specify cortical and calvarial bone development in juvenile mice

The cortical bone showed a positive enrichment for gene sets related to DNA including its replication, homologous recombination, mismatch repair as well as nucleotide excision repair and metabolism. Wang and Li, (75) summarized how the expression of DNA damage and cell cycle associated genes regulate *Sp7* during osteoblast differentiation, further suggesting that as only osteoblasts express *Sp7*, this connection was not observed in other cell types. Moreover, the low bone mass associated diseases Werner’s syndrome and Hutchinson Gilford Progeria syndrome are caused respectively by loss of the DNA repair enzyme *WRN RecQ like helicase* (*Wrn*) or the nuclear matrix protein *Lmna* (76). Considering the enrichment of several DNA related gene sets between cortices and calvaria as well as that most bone inhabiting cells are post-mitotic osteocytes (77), further studies on intramembranous bones should elucidate the relevance of the cell cycle genes during bone modeling.

Finally, the cortical bone exhibited a positive enrichment for gene sets associated with ribosome biogenesis in the GSEA. The occurrence of site-specific bone diseases related to ribosome associated genes suggests that despite the universality of ribosomes, different bone tissues have a ribosome machinery of diverging composition fitting the tissue’s function (78). For example, RPL10 has been detected in several regions of the developing bovine femur, mostly in cells that are poised to produce a mineralized matrix and its expression diminishing when the mineralization complex has been established. RPL10 was found in the perichondrium and periosteum surrounding the growth plate but not in osteocytes (79). This indicates that the non-removal of the periosteum could have affected the differential enrichment of the ribosome gene set in this study. The site-specific ribosomal function during bone formation could be mediated by various interaction partners such as the osteogenic master regulator RUNX2 (60). Furthermore, Treacher-Collins syndrome, a disease affecting only the cranial bone formation but not the rest of the skeleton, is caused by mutations in the ribosomal DNA transcription regulator *Tcof1*, despite the universal expression of *Tcof1* in embryonic and adult tissues (80). Other studies link the processes of ribosome biogenesis, cell cycle arrest, increased cell death and reduced proliferation and migration of neural crest cells, a cell population strongly involved in cranial bone formation (81, 82). Deletion of *Tcof1* in murine neuroblastoma cell line changed the expression pattern of genes associated with various processes, such as cell cycle and development, beyond ribosomal DNA regulation (83). We hypothesize that the differential enrichment of KEGG gene sets related to cell cycle, DNA damage and ribosome biogenesis suggest a precise mechanism for diverging bone development despite the universal expression of, e.g., cell cycle-associated genes. Furthermore, the differential enrichment of these pathway terms indicates the involvement of the cranial neural crest as an origin for the observed differences in gene expression between both bones. In the first step, the involvement of the cranial neural crest already specifies the developmental program of the calvaria, despite both calvaria and cortical bone develop through the mechanism of intramembranous ossification. Subsequently, the modulation of signaling pathways like WNT according to the bones origin participate in forming bone-specific differences.

### The comparison between juvenile and mature calvaria and cortices reveals a continuous developmental program

We investigated age-related changes of differential gene expression between cortical bone and calvaria by comparing our list of DEGs from juvenile mice and the DEGs reported by Wang et al. (16) and Rawlinson et al. (17) from mature animals. First, we found a high number of bone-associated DEGs having the same expression pattern in juvenile mice and mature animals, such as genes related to patterning, WNT signaling and craniosynostosis. Several other genes classified as DEGs in adult bones by Wang et al. (2020) (16) and Rawlinson et al. (17), such as *Wnt16*, *Fibroblast growth factor 1* (*Fgf1*), *Dkk1*, *Sost* and a few *Hox* genes were not found to be differentially expressed in juvenile bones in our study. In addition to the age differences, this may be attributed to our samples’ origin from female mice compared to male rats being investigated by Rawlinson et al. (17). Moreover, contrary to Wang et al. (16), we collected the samples from the femur, not from the tibia, and we did not remove the periosteum and cranial sutures. As discussed earlier, this may significantly affect DEG detection, considering these tissues’ functions of harboring stem cell populations and expressing signaling molecules (18). DEGs detected only in this study and have not been found by Wang et al. (16) and Rawlinson et al. (17), therefore, this can only indicate a juvenile specific gene set, but still needs to be verified in subsequent studies. However, it is unlikely that non-removal of sutures and periosteum affects the differential expression analysis of bone cell restricted genes such as *Bglap* (84). Considering the younger age of our mice, processes such as bone growth (12) and the expression level of associated genes will therefore differ from mature animals. However, we cannot exclude that some of the DEGs that were only found in our study on juvenile animals, would also be considered differentially expressed by Wang et al. (16), if the same threshold was applied and their analysis was limited to DEGs between murine calvaria and tibia samples (n=2) instead of only including DEGs showing the same trend between calvaria and tibia in mice, rats and macaques.

We suggest that during the juvenile and mature phase, differences in bone (re)modeling of cortical and calvarial bone could primarily be mediated by a key gene set related to pathways like BMP, HH and WNT signaling. Many additional genes related to these pathways only show a differential expression between the cortices and calvaria either in juvenile animals during bone modeling or mature animals during remodeling. In line with this is our finding of only four genes *Mepe*, *Acp5*, *Cspr3* and *Cdh15* showing an opposing expression pattern between juvenile mice and mature animals.

In conclusion, a high number of DEGs detected between juvenile murine calvaria and cortices were also found in mature animals, whereas only four genes showed an opposing expression pattern between juvenile and mature samples. This indicates a DEG key set, that is independent of age is constantly differentially expressed between the cortical and calvarial bone. Thus, determining the character of both bones’ structure and function. For example, the high expression levels of *Lox* in the cortical bone provide the bone’s stiffness for locomotion in juvenile and mature mice, as locomotion is essential age-independently. This is complemented by age-dependent DEGs as demonstrated in this study or reported by Wang et al. (16) and Rawlinson et al. (17). For example, the calcification associated gene *Bglap* was only differentially expressed in juvenile mice. Many other age-dependent DEGs are related to signaling pathways and future studies will help to reveal how their expression is linked to the development of bone-site specific differences.

### Bone-site specific phenotype development

The prediction how differential gene expression affects bone development in this and other published studies is strongly deterred by the bone-site specific function of genes. For example, *Msx1* exhibited a significantly higher expression level in the calvaria compared to the cortices of juvenile and adult mice (17), and it’s expression has been linked to directional craniofacial bone growth. *Msx1* affects the mandibular trabecular and mandibular cortical bone in a different manner (85). Murine *Msx1* overexpression using a Col1a promoter showed a higher collagenous bone matrix volume in the trabecular mandibles that was less mineralized because of limited osteocyte differentiation in the skull. Contrary to this, the overexpression of *Msx1* led to increased apoptosis in the cortical mandibles, suggesting site-specific effects of *Msx1*.

Next, contrary to the study of Wang et al. (16) on adult animals, in our study *Mepe* expression level was higher in the juvenile calvaria compared to the cortices. A knock out of *Mepe* was reported to decrease calvarial bone mineralization (86) and on the other hand to increase osteoblast number and trabecular bone mass *in vivo* as well as to increase mineralized nodules in osteoblast cell culture (87). Since both studies performed a *Mepe* knock out and did not investigate a decreased *Mepe* expression level, it is difficult to transfer their insights to Wang et al. (16) and to our study. In line with Gullard et al. (86) and Gowen et al. (87), the higher expression of *Mepe* in juvenile mice undergoing bone modeling as demonstrated in our study could indicate a site-specific effect of Mepe allowing for quick calvarial mineralization and long bone osteoblast proliferation. In the adult animal, a lower expression level of *Mepe* in the calvaria may inhibit ectopic calcification whereas the higher expression level of *Mepe* in the long bone decreases osteoblast numbers to a minimum that is sufficient for bone remodeling.

It should be considered that additional bone related DEGs may exhibit such a site-specific effect between the calvaria and the long bones, adding another regulatory level of bone development and highlighting the importance of subsequent experiments to clarify the genes function in each bone site.

## Conclusion

This study, based on a high number of biological replicates from freshly isolated tissue, compared the transcriptome of two bone types that share a similar developmental ontogeny of membranous ossification but display a highly different shape and function. Our data revealed for the first-time significant differences between the transcriptome of the calvaria and cortical bone in juvenile female mice. Our results differed from previous studies about calvarial and cortical bone homeostasis in adult animals, highlighting the most crucial pathway mediators for juvenile skeletal development. However, a core set of DEGs was found in juvenile mice and mature animals. The differentially expressed genes detected in our study were related to bone diseases, craniosynostosis and weight loading. Intriguingly the expression level of most weight-loading associated genes was higher in the calvaria than in the cortical bone. This could be related to a mechanism preventing bone loss in the non-weight-bearing calvaria. Moreover, we confirmed the widely known importance of signaling pathways like WNT and HH for bone development while identifying new potential candidates participating in site-specific regulation of bone development. Using GSEA, we demonstrated that the calvaria exhibited a positive enrichment for signaling pathways. In contrast, the cortical bone was positively enriched for gene sets related to DNA replication, cell cycle and ribosome.

## Data availability statement

The datasets presented in this study can be found in online repositories. The names of the repository/repositories and accession number(s) can be found below: https://www.ncbi.nlm.nih.gov/geo/query/acc.cgi?acc=GSE223750.

## Ethics statement

The animal study was reviewed and approved by the Hebrew University Animal Care Committee (license number 21-16657-3).

## Author contributions

JJ, RK-A, DS-D and EM-O contributed to conception and design of the study. JJ and TS organized the database. JJ and TS performed the statistical analysis. JJ wrote the first draft of the manuscript. JJ and RK-A wrote sections of the manuscript. All authors contributed to the article and approved the submitted version.

## Supplementary material

Supplementary Material Figure 1 Enriched KEGG gene sets determined by GSEA clusterProfiler and PathView. **(A)** Wntsignaling pathway, **(B)** Hedgehog signaling pathway, **(C)** Ribosome, **(D)** Ribosome biogenesis in eukaryotes and **(E)** ECM-receptor interaction. Colors indicate normalized gene expression change value between cortices (1) and calvaria (-1).
