# Supplementary figures and images for: Differential gene expression in the calvarial and cortical bone of juvenile female mice
Source: Front Endocrinol (Lausanne). 2023 Jun 12;14:1127536. doi: 10.3389/fendo.2023.1127536 (PMC10291685; doi:10.3389/fendo.2023.1127536)

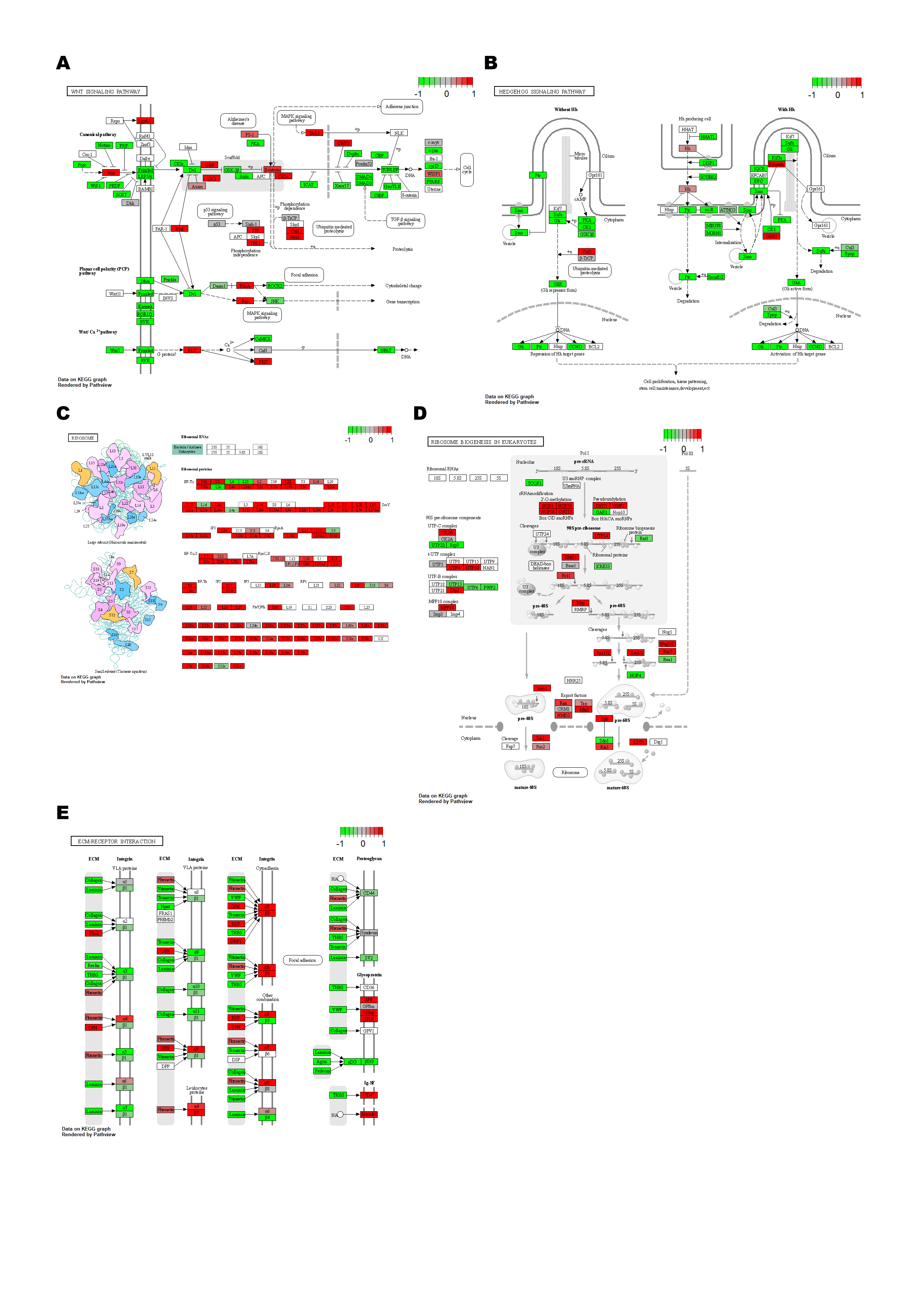

Supplement: Supplementary Material Figure 1 — Enriched KEGG gene sets determined by GSEA clusterProfiler and PathView. (A) Wntsignaling pathway, (B) Hedgehog signaling pathway, (C) Ribosome, (D) Ribosome biogenesis in eukaryotes and (E) ECM-receptor interaction. Colors indicate normalized gene expression change value between cortices (1) and calvaria (-1). [file Image_1.png]
