# Supplementary material for: Differential gene expression in the calvarial and cortical bone of juvenile female mice
Source: Front Endocrinol (Lausanne). 2023 Jun 12;14:1127536. doi: 10.3389/fendo.2023.1127536 (PMC10291685; doi:10.3389/fendo.2023.1127536)
Supplement: Supplementary file 2 [file Table_1.docx]

**Supplemental material**

**Table S1:** Top 100 expressed genes in cortices and calvaria

|  |  | **Cortices** |  |  | **Calvaria** |  |
| --- | --- | --- | --- | --- | --- | --- |
|  | Gene ID | Gene name | Avg count | Gene ID | Gene name | Avg count |
| 1 | ENSMUSG00000052305 | Hbb-bs | 39138 | ENSMUSG00000052305 | Hbb-bs | 47348 |
| 2 | ENSMUSG00000069919 | Hba-a1 | 20388 | ENSMUSG00000069919 | Hba-a1 | 21204 |
| 3 | ENSMUSG00000029661 | Col1a2 | 20078 | ENSMUSG00000029661 | Col1a2 | 16703 |
| 4 | ENSMUSG00000073940 | Hbb-bt | 13739 | ENSMUSG00000073940 | Hbb-bt | 16093 |
| 5 | ENSMUSG00000018593 | Sparc | 11899 | ENSMUSG00000018593 | Sparc | 10416 |
| 6 | ENSMUSG00000001506 | Col1a1 | 10404 | ENSMUSG00000001506 | Col1a1 | 9879 |
| 7 | ENSMUSG00000074483 | Bglap | 5513 | ENSMUSG00000069917 | Hba-a2 | 5143 |
| 8 | ENSMUSG00000029580 | Actb | 4378 | ENSMUSG00000029580 | Actb | 4257 |
| 9 | ENSMUSG00000007892 | Rplp1 | 3997 | ENSMUSG00000007892 | Rplp1 | 3908 |
| 10 | ENSMUSG00000069917 | Hba-a2 | 2936 | ENSMUSG00000106106 | CT010467.1 | 2643 |
| 11 | ENSMUSG00000001348 | Acp5 | 2751 | ENSMUSG00000031972 | Acta1 | 2613 |
| 12 | ENSMUSG00000106106 | CT010467.1 | 2647 | ENSMUSG00000074483 | Bglap | 2529 |
| 13 | ENSMUSG00000032518 | Rpsa | 2545 | ENSMUSG00000063856 | Gpx1 | 2514 |
| 14 | ENSMUSG00000067274 | Rplp0 | 2538 | ENSMUSG00000032518 | Rpsa | 2273 |
| 15 | ENSMUSG00000063856 | Gpx1 | 2490 | ENSMUSG00000067274 | Rplp0 | 2268 |
| 16 | ENSMUSG00000074486 | Bglap2 | 2436 | ENSMUSG00000047675 | Rps8 | 2075 |
| 17 | ENSMUSG00000047675 | Rps8 | 2207 | ENSMUSG00000018339 | Gpx3 | 1995 |
| 18 | ENSMUSG00000003970 | Rpl8 | 2044 | ENSMUSG00000026043 | Col3a1 | 1990 |
| 19 | ENSMUSG00000032399 | Rpl4 | 2027 | ENSMUSG00000061983 | Rps12 | 1872 |
| 20 | ENSMUSG00000024608 | Rps14 | 2013 | ENSMUSG00000024608 | Rps14 | 1870 |
| 21 | ENSMUSG00000012848 | Rps5 | 1959 | ENSMUSG00000003970 | Rpl8 | 1867 |
| 22 | ENSMUSG00000038357 | Camp | 1914 | ENSMUSG00000001348 | Acp5 | 1795 |
| 23 | ENSMUSG00000045128 | Rpl18a | 1905 | ENSMUSG00000030399 | Ckm | 1748 |
| 24 | ENSMUSG00000030744 | Rps3 | 1899 | ENSMUSG00000012848 | Rps5 | 1719 |
| 25 | ENSMUSG00000008668 | Rps18 | 1867 | ENSMUSG00000045128 | Rpl18a | 1716 |
| 26 | ENSMUSG00000020372 | Rack1 | 1861 | ENSMUSG00000008668 | Rps18 | 1599 |
| 27 | ENSMUSG00000093674 | Rpl41 | 1847 | ENSMUSG00000040952 | Rps19 | 1524 |
| 28 | ENSMUSG00000061983 | Rps12 | 1767 | ENSMUSG00000030744 | Rps3 | 1514 |
| 29 | ENSMUSG00000040952 | Rps19 | 1672 | ENSMUSG00000032399 | Rpl4 | 1499 |
| 30 | ENSMUSG00000044533 | Rps2 | 1670 | ENSMUSG00000093674 | Rpl41 | 1471 |
| 31 | ENSMUSG00000039001 | Rps21 | 1600 | ENSMUSG00000020372 | Rack1 | 1467 |
| 32 | ENSMUSG00000025362 | Rps26 | 1558 | ENSMUSG00000024661 | Fth1 | 1441 |
| 33 | ENSMUSG00000028234 | Rps20 | 1544 | ENSMUSG00000031375 | Bgn | 1426 |
| 34 | ENSMUSG00000034892 | Rps29 | 1544 | ENSMUSG00000025362 | Rps26 | 1418 |
| 35 | ENSMUSG00000000740 | Rpl13 | 1532 | ENSMUSG00000044533 | Rps2 | 1412 |
| 36 | ENSMUSG00000025794 | Rpl14 | 1506 | ENSMUSG00000039001 | Rps21 | 1401 |
| 37 | ENSMUSG00000063457 | Rps15 | 1414 | ENSMUSG00000000753 | Serpinf1 | 1400 |
| 38 | ENSMUSG00000027562 | Car2 | 1391 | ENSMUSG00000018893 | Mb | 1399 |
| 39 | ENSMUSG00000038900 | Rpl12 | 1368 | ENSMUSG00000019505 | Ubb | 1386 |
| 40 | ENSMUSG00000019505 | Ubb | 1360 | ENSMUSG00000061723 | Tnnt3 | 1363 |
| 41 | ENSMUSG00000024661 | Fth1 | 1323 | ENSMUSG00000000740 | Rpl13 | 1362 |
| 42 | ENSMUSG00000023004 | Tuba1b | 1298 | ENSMUSG00000034892 | Rps29 | 1290 |
| 43 | ENSMUSG00000059291 | Rpl11 | 1284 | ENSMUSG00000025794 | Rpl14 | 1282 |
| 44 | ENSMUSG00000061477 | Rps7 | 1246 | ENSMUSG00000034994 | Eef2 | 1281 |
| 45 | ENSMUSG00000031375 | Bgn | 1233 | ENSMUSG00000074486 | Bglap2 | 1278 |
| 46 | ENSMUSG00000032484 | Ngp | 1206 | ENSMUSG00000017300 | Tnnc2 | 1255 |
| 47 | ENSMUSG00000034994 | Eef2 | 1191 | ENSMUSG00000063457 | Rps15 | 1255 |
| 48 | ENSMUSG00000025508 | Rplp2 | 1189 | ENSMUSG00000030672 | Mylpf | 1252 |
| 49 | ENSMUSG00000058546 | Rpl23a | 1182 | ENSMUSG00000028234 | Rps20 | 1235 |
| 50 | ENSMUSG00000041841 | Rpl37 | 1158 | ENSMUSG00000031097 | Tnni2 | 1194 |
| 51 | ENSMUSG00000028111 | Ctsk | 1151 | ENSMUSG00000025508 | Rplp2 | 1187 |
| 52 | ENSMUSG00000005161 | Prdx2 | 1148 | ENSMUSG00000023004 | Tuba1b | 1136 |
| 53 | ENSMUSG00000057322 | Rpl38 | 1093 | ENSMUSG00000038900 | Rpl12 | 1121 |
| 54 | ENSMUSG00000069516 | Lyz2 | 1092 | ENSMUSG00000006574 | Slc4a1 | 1119 |
| 55 | ENSMUSG00000062328 | Rpl17 | 1088 | ENSMUSG00000027562 | Car2 | 1088 |
| 56 | ENSMUSG00000057841 | Rpl32 | 1078 | ENSMUSG00000038357 | Camp | 1077 |
| 57 | ENSMUSG00000024330 | Col11a2 | 1077 | ENSMUSG00000005161 | Prdx2 | 1027 |
| 58 | ENSMUSG00000046330 | Rpl37a | 1077 | ENSMUSG00000057322 | Rpl38 | 1020 |
| 59 | ENSMUSG00000000753 | Serpinf1 | 1066 | ENSMUSG00000059291 | Rpl11 | 1013 |
| 60 | ENSMUSG00000071415 | Rpl23 | 1053 | ENSMUSG00000070436 | Serpinh1 | 1012 |
| 61 | ENSMUSG00000062647 | Rpl7a | 1050 | ENSMUSG00000046330 | Rpl37a | 1009 |
| 62 | ENSMUSG00000030432 | Rpl28 | 1049 | ENSMUSG00000027447 | Cst3 | 1007 |
| 63 | ENSMUSG00000022483 | Col2a1 | 1032 | ENSMUSG00000027523 | Gnas | 984 |
| 64 | ENSMUSG00000006574 | Slc4a1 | 1021 | ENSMUSG00000061477 | Rps7 | 983 |
| 65 | ENSMUSG00000060938 | Rpl26 | 994 | ENSMUSG00000041841 | Rpl37 | 979 |
| 66 | ENSMUSG00000056201 | Cfl1 | 991 | ENSMUSG00000092341 | Malat1 | 971 |
| 67 | ENSMUSG00000015656 | Hspa8 | 984 | ENSMUSG00000030432 | Rpl28 | 945 |
| 68 | ENSMUSG00000029304 | Spp1 | 980 | ENSMUSG00000062328 | Rpl17 | 930 |
| 69 | ENSMUSG00000062006 | Rpl34 | 962 | ENSMUSG00000058546 | Rpl23a | 922 |
| 70 | ENSMUSG00000047215 | Rpl9 | 952 | ENSMUSG00000032366 | Tpm1 | 906 |
| 71 | ENSMUSG00000028081 | Rps3a1 | 950 | ENSMUSG00000015656 | Hspa8 | 898 |
| 72 | ENSMUSG00000027523 | Gnas | 936 | ENSMUSG00000028111 | Ctsk | 889 |
| 73 | ENSMUSG00000057863 | Rpl36 | 927 | ENSMUSG00000000031 | H19 | 877 |
| 74 | ENSMUSG00000031972 | Acta1 | 923 | ENSMUSG00000023944 | Hsp90ab1 | 874 |
| 75 | ENSMUSG00000070436 | Serpinh1 | 911 | ENSMUSG00000024330 | Col11a2 | 859 |
| 76 | ENSMUSG00000060036 | Rpl3 | 905 | ENSMUSG00000056201 | Cfl1 | 850 |
| 77 | ENSMUSG00000006333 | Rps9 | 901 | ENSMUSG00000071415 | Rpl23 | 850 |
| 78 | ENSMUSG00000059070 | Rpl18 | 856 | ENSMUSG00000006333 | Rps9 | 844 |
| 79 | ENSMUSG00000067288 | Rps28 | 834 | ENSMUSG00000025270 | Alas2 | 820 |
| 80 | ENSMUSG00000078812 | Eif5a | 814 | ENSMUSG00000060938 | Rpl26 | 812 |
| 81 | ENSMUSG00000027447 | Cst3 | 809 | ENSMUSG00000057841 | Rpl32 | 809 |
| 82 | ENSMUSG00000073702 | Rpl31 | 788 | ENSMUSG00000067288 | Rps28 | 799 |
| 83 | ENSMUSG00000041453 | Rpl21 | 775 | ENSMUSG00000062647 | Rpl7a | 799 |
| 84 | ENSMUSG00000023944 | Hsp90ab1 | 774 | ENSMUSG00000029304 | Spp1 | 796 |
| 85 | ENSMUSG00000038274 | Fau | 773 | ENSMUSG00000057863 | Rpl36 | 786 |
| 86 | ENSMUSG00000017737 | Mmp9 | 734 | ENSMUSG00000028464 | Tpm2 | 780 |
| 87 | ENSMUSG00000022108 | Itm2b | 724 | ENSMUSG00000062006 | Rpl34 | 773 |
| 88 | ENSMUSG00000036371 | Serbp1 | 724 | ENSMUSG00000028081 | Rps3a1 | 772 |
| 89 | ENSMUSG00000090862 | Rps13 | 716 | ENSMUSG00000069516 | Lyz2 | 759 |
| 90 | ENSMUSG00000003814 | Calr | 714 | ENSMUSG00000068220 | Lgals1 | 757 |
| 91 | ENSMUSG00000035242 | Oaz1 | 713 | ENSMUSG00000030695 | Aldoa | 755 |
| 92 | ENSMUSG00000060126 | Tpt1 | 711 | ENSMUSG00000078812 | Eif5a | 745 |
| 93 | ENSMUSG00000030399 | Ckm | 707 | ENSMUSG00000003814 | Calr | 742 |
| 94 | ENSMUSG00000057729 | Prtn3 | 704 | ENSMUSG00000035242 | Oaz1 | 742 |
| 95 | ENSMUSG00000048758 | Rpl29 | 699 | ENSMUSG00000031818 | Cox4i1 | 738 |
| 96 | ENSMUSG00000031818 | Cox4i1 | 691 | ENSMUSG00000060036 | Rpl3 | 718 |
| 97 | ENSMUSG00000025130 | P4hb | 689 | ENSMUSG00000047215 | Rpl9 | 710 |
| 98 | ENSMUSG00000025393 | Atp5b | 688 | ENSMUSG00000025393 | Atp5b | 706 |
| 99 | ENSMUSG00000061787 | Rps17 | 683 | ENSMUSG00000032562 | Gnai2 | 702 |
| 100 | ENSMUSG00000056054 | S100a8 | 678 | ENSMUSG00000025130 | P4hb | 693 |

**Table S2:** Expression level of several bone relevant genes

|  | Gene ID | Gene name | Avg counts in cortices | Avg counts in calvaria |
| --- | --- | --- | --- | --- |
| Bone and carilage matrix | ENSMUSG00000030607 | Acan | 98.69 | 74.53 |
|  | ENSMUSG00000001348 | Acp5 | 2750.53 | 1794.72 |
|  | ENSMUSG00000028766 | Alpl | 439.48 | 431.66 |
|  | ENSMUSG00000074483 | Bglap | 5513.35 | 2529.10 |
|  | ENSMUSG00000074486 | Bglap2 | 2435.58 | 1278.33 |
|  | ENSMUSG00000074489 | Bglap3 | 32.02 | 15.56 |
|  | ENSMUSG00000027562 | Car2 | 1390.76 | 1087.65 |
|  | ENSMUSG00000039462 | Col10a1 | 138.34 | 91.27 |
|  | ENSMUSG00000027966 | Col11a1 | 571.84 | 163.26 |
|  | ENSMUSG00000024330 | Col11a2 | 1077.46 | 859.15 |
|  | ENSMUSG00000001506 | Col1a1 | 10404.21 | 9879.18 |
|  | ENSMUSG00000029661 | Col1a2 | 20077.61 | 16702.55 |
|  | ENSMUSG00000022483 | Col2a1 | 1031.76 | 362.79 |
|  | ENSMUSG00000026147 | Col9a1 | 46.92 | 25.94 |
|  | ENSMUSG00000028626 | Col9a2 | 71.66 | 42.76 |
|  | ENSMUSG00000027570 | Col9a3 | 74.15 | 41.63 |
|  | ENSMUSG00000031849 | Comp | 104.81 | 78.35 |
|  | ENSMUSG00000040314 | Ctsg | 217.25 | 71.51 |
|  | ENSMUSG00000028111 | Ctsk | 1151.40 | 888.92 |
|  | ENSMUSG00000029307 | Dmp1 | 174.76 | 116.75 |
|  | ENSMUSG00000026193 | Fn1 | 262.83 | 246.30 |
|  | ENSMUSG00000029306 | Ibsp | 422.44 | 283.34 |
|  | ENSMUSG00000020583 | Matn3 | 24.80 | 8.64 |
|  | ENSMUSG00000053863 | Mepe | 102.80 | 226.42 |
|  | ENSMUSG00000030218 | Mgp | 313.07 | 613.89 |
|  | ENSMUSG00000050578 | Mmp13 | 9.27 | 7.11 |
|  | ENSMUSG00000000957 | Mmp14 | 322.78 | 527.44 |
|  | ENSMUSG00000017737 | Mmp9 | 733.76 | 540.77 |
|  | ENSMUSG00000048450 | Msx1 | 5.73 | 30.79 |
|  | ENSMUSG00000022952 | Runx1 | 51.18 | 49.47 |
|  | ENSMUSG00000039153 | Runx2 | 43.54 | 65.46 |
|  | ENSMUSG00000070691 | Runx3 | 16.62 | 13.25 |
|  | ENSMUSG00000027833 | Shox2 | 23.22 | 2.44 |
|  | ENSMUSG00000041540 | Sox5 | 1.45 | 2.72 |
|  | ENSMUSG00000051910 | Sox6 | 24.74 | 33.51 |
|  | ENSMUSG00000000567 | Sox9 | 3.56 | 2.01 |
|  | ENSMUSG00000060284 | Sp7 | 64.88 | 53.92 |
|  | ENSMUSG00000018593 | Sparc | 11898.52 | 10415.53 |
|  | ENSMUSG00000001131 | Timp1 | 141.31 | 158.01 |
|  | ENSMUSG00000017466 | Timp2 | 278.68 | 545.32 |
|  | ENSMUSG00000020044 | Timp3 | 50.91 | 90.50 |
| BMP/TGF | ENSMUSG00000026836 | Acvr1 | 15.99 | 19.58 |
|  | ENSMUSG00000000532 | Acvr1b | 10.47 | 12.39 |
|  | ENSMUSG00000061393 | Acvr2b | 6.51 | 10.73 |
|  | ENSMUSG00000022098 | Bmp1 | 271.89 | 241.76 |
|  | ENSMUSG00000027358 | Bmp2 | 4.90 | 5.99 |
|  | ENSMUSG00000021835 | Bmp4 | 23.64 | 30.49 |
|  | ENSMUSG00000039004 | Bmp6 | 7.21 | 8.13 |
|  | ENSMUSG00000032726 | Bmp8a | 6.52 | 13.35 |
|  | ENSMUSG00000048616 | Nog | 1.95 | 9.96 |
|  | ENSMUSG00000031681 | Smad1 | 7.63 | 6.76 |
|  | ENSMUSG00000024563 | Smad2 | 6.14 | 4.58 |
|  | ENSMUSG00000032402 | Smad3 | 21.81 | 29.22 |
|  | ENSMUSG00000024515 | Smad4 | 29.14 | 39.59 |
|  | ENSMUSG00000021540 | Smad5 | 25.89 | 17.70 |
|  | ENSMUSG00000036867 | Smad6 | 38.68 | 38.26 |
|  | ENSMUSG00000025880 | Smad7 | 23.81 | 29.37 |
|  | ENSMUSG00000038780 | Smurf1 | 12.08 | 16.86 |
|  | ENSMUSG00000018363 | Smurf2 | 36.11 | 41.71 |
|  | ENSMUSG00000002603 | Tgfb1 | 221.10 | 220.35 |
|  | ENSMUSG00000039239 | Tgfb2 | 10.63 | 21.88 |
|  | ENSMUSG00000021253 | Tgfb3 | 35.70 | 75.19 |
|  | ENSMUSG00000007613 | Tgfbr1 | 45.33 | 34.24 |
|  | ENSMUSG00000029287 | Tgfbr3 | 6.06 | 8.59 |
|  | ENSMUSG00000006932 | Ctnnb1 | 283.55 | 259.79 |
| Wnt | ENSMUSG00000044548 | Dact1 | 3.88 | 10.01 |
|  | ENSMUSG00000078794 | Dact3 | 6.77 | 15.10 |
|  | ENSMUSG00000024868 | Dkk1 | 14.03 | 13.62 |
|  | ENSMUSG00000030772 | Dkk3 | 29.40 | 72.38 |
|  | ENSMUSG00000029071 | Dvl1 | 40.53 | 60.59 |
|  | ENSMUSG00000027004 | Frzb | 8.20 | 2.00 |
|  | ENSMUSG00000044674 | Fzd1 | 6.16 | 25.63 |
|  | ENSMUSG00000040249 | Lrp1 | 184.79 | 356.53 |
|  | ENSMUSG00000027253 | Lrp4 | 35.03 | 60.00 |
|  | ENSMUSG00000024913 | Lrp5 | 36.30 | 53.29 |
|  | ENSMUSG00000030201 | Lrp6 | 19.49 | 31.37 |
|  | ENSMUSG00000021464 | Ror2 | 5.83 | 22.52 |
|  | ENSMUSG00000027996 | Sfrp2 | 7.41 | 135.49 |
|  | ENSMUSG00000050010 | Shisa3 | 1.05 | 8.39 |
|  | ENSMUSG00000001494 | Sost | 30.60 | 57.37 |
|  | ENSMUSG00000055799 | Tcf7l1 | 22.50 | 35.81 |
|  | ENSMUSG00000035799 | Twist1 | 42.69 | 135.74 |
|  | ENSMUSG00000022996 | Wnt10b | 2.10 | 4.17 |
|  | ENSMUSG00000015957 | Wnt11 | 1.73 | 5.49 |
|  | ENSMUSG00000029671 | Wnt16 | 5.60 | 2.49 |
|  | ENSMUSG00000036856 | Wnt4 | 6.87 | 2.35 |
|  | ENSMUSG00000021994 | Wnt5a | 6.64 | 6.01 |
|  | ENSMUSG00000030170 | Wnt5b | 19.76 | 28.05 |
|  | ENSMUSG00000022382 | Wnt7b | 1.69 | 3.29 |
|  | ENSMUSG00000000126 | Wnt9a | 1.54 | 6.28 |
| Ihh | ENSMUSG00000030323 | Ift122 | 5.77 | 12.39 |
|  | ENSMUSG00000006538 | Ihh | 6.50 | 5.68 |
|  | ENSMUSG00000001761 | Smo | 20.43 | 27.40 |
|  | ENSMUSG00000021466 | Ptch1 | 9.61 | 12.87 |
|  | ENSMUSG00000025407 | Gli1 | 6.48 | 21.61 |
|  | ENSMUSG00000048402 | Gli2 | 3.37 | 3.49 |
|  | ENSMUSG00000021318 | Gli3 | 4.47 | 3.01 |
|  | ENSMUSG00000032492 | Pth1r | 214.89 | 263.89 |

**Table S3:** Differentially expressed genes

| Gene ID | Gene name | log2FC (Cortices/Calvaria) | pad.j |
| --- | --- | --- | --- |
| ENSMUSG00000075588 | Hoxb2 | 4.97 | 0.00E+00 |
| ENSMUSG00000001661 | Hoxc6 | 4.11 | 4.95E-07 |
| ENSMUSG00000021506 | Pitx1 | 4.09 | 1.15E-08 |
| ENSMUSG00000000938 | Hoxa10 | 3.93 | 1.12E-06 |
| ENSMUSG00000024681 | Ms4a3 | 3.17 | 1.94E-03 |
| ENSMUSG00000027833 | Shox2 | 3.17 | 0.00E+00 |
| ENSMUSG00000060459 | Kng2 | 2.93 | 1.84E-03 |
| ENSMUSG00000038236 | Hoxa7 | 2.85 | 5.19E-08 |
| ENSMUSG00000022875 | Kng1 | 2.76 | 2.45E-04 |
| ENSMUSG00000038227 | Hoxa9 | 2.74 | 5.84E-06 |
| ENSMUSG00000081792 | Anp32b-ps1 | 2.57 | 5.00E-02 |
| ENSMUSG00000024205 | Rpl36-ps2 | 2.55 | 2.05E-02 |
| ENSMUSG00000038560 | Sp6 | 2.47 | 5.93E-03 |
| ENSMUSG00000032373 | Car12 | 2.36 | 4.30E-07 |
| ENSMUSG00000068129 | Cst7 | 2.11 | 5.07E-03 |
| ENSMUSG00000027004 | Frzb | 1.98 | 1.67E-02 |
| ENSMUSG00000027966 | Col11a1 | 1.80 | 0.00E+00 |
| ENSMUSG00000004612 | Nkg7 | 1.67 | 1.16E-04 |
| ENSMUSG00000027800 | Tm4sf1 | 1.66 | 4.91E-02 |
| ENSMUSG00000022303 | Dcstamp | 1.63 | 1.11E-02 |
| ENSMUSG00000040314 | Ctsg | 1.61 | 2.23E-05 |
| ENSMUSG00000046814 | Gchfr | 1.60 | 1.56E-02 |
| ENSMUSG00000027559 | Car3 | 1.58 | 0.00E+00 |
| ENSMUSG00000027698 | Nceh1 | 1.58 | 3.37E-02 |
| ENSMUSG00000025014 | Dntt | 1.53 | 3.05E-03 |
| ENSMUSG00000012519 | Mlkl | 1.51 | 2.54E-02 |
| ENSMUSG00000056486 | Chn1 | 1.50 | 2.06E-05 |
| ENSMUSG00000041608 | Entpd3 | 1.49 | 7.89E-05 |
| ENSMUSG00000045248 | Med26 | 1.49 | 2.61E-02 |
| ENSMUSG00000032374 | Plod2 | 1.48 | 1.57E-06 |
| ENSMUSG00000040809 | Chil3 | 1.44 | 4.78E-02 |
| ENSMUSG00000050751 | Pgbd5 | 1.44 | 1.57E-02 |
| ENSMUSG00000029322 | Plac8 | 1.43 | 4.88E-02 |
| ENSMUSG00000038301 | Snx10 | 1.42 | 3.54E-02 |
| ENSMUSG00000017697 | Ada | 1.41 | 9.20E-03 |
| ENSMUSG00000050147 | F2rl3 | 1.40 | 4.88E-02 |
| ENSMUSG00000020652 | Cenpo | 1.39 | 1.24E-02 |
| ENSMUSG00000024990 | Rbp4 | 1.37 | 1.44E-02 |
| ENSMUSG00000031578 | Mak16 | 1.32 | 4.99E-02 |
| ENSMUSG00000019810 | Fuca2 | 1.31 | 1.57E-02 |
| ENSMUSG00000031562 | Dctd | 1.29 | 2.63E-02 |
| ENSMUSG00000026826 | Nr4a2 | 1.28 | 4.39E-02 |
| ENSMUSG00000004791 | Pgf | 1.25 | 1.53E-02 |
| ENSMUSG00000027170 | Eif3m | 1.25 | 2.94E-02 |
| ENSMUSG00000026835 | Fcnb | 1.24 | 1.81E-02 |
| ENSMUSG00000022584 | Ly6c2 | 1.20 | 2.05E-02 |
| ENSMUSG00000041481 | Serpina3g | 1.20 | 3.29E-04 |
| ENSMUSG00000029275 | Gfi1 | 1.18 | 4.39E-02 |
| ENSMUSG00000033491 | Prss35 | 1.18 | 7.34E-04 |
| ENSMUSG00000033022 | Cdo1 | 1.17 | 5.07E-03 |
| ENSMUSG00000050953 | Gja1 | 1.17 | 8.40E-04 |
| ENSMUSG00000024640 | Psat1 | 1.15 | 8.84E-04 |
| ENSMUSG00000033998 | Kcnk1 | 1.15 | 1.57E-02 |
| ENSMUSG00000020805 | Slc13a5 | 1.14 | 3.67E-03 |
| ENSMUSG00000009350 | Mpo | 1.12 | 1.51E-03 |
| ENSMUSG00000074483 | Bglap | 1.12 | 5.58E-06 |
| ENSMUSG00000012483 | Rpa3 | 1.11 | 4.21E-02 |
| ENSMUSG00000057729 | Prtn3 | 1.09 | 5.95E-03 |
| ENSMUSG00000080198 | Rpl10a-ps4 | 1.09 | 3.26E-02 |
| ENSMUSG00000020125 | Elane | 1.08 | 5.93E-03 |
| ENSMUSG00000060470 | Adgrg3 | 1.05 | 1.45E-03 |
| ENSMUSG00000075370 | Igll1 | 1.03 | 2.41E-02 |
| ENSMUSG00000045319 | Proser2 | 1.02 | 3.13E-02 |
| ENSMUSG00000029484 | Anxa3 | 1.01 | 1.53E-02 |
| ENSMUSG00000021186 | Fbln5 | 1.00 | 7.24E-04 |
| ENSMUSG00000022221 | Ripk3 | 0.99 | 4.75E-03 |
| ENSMUSG00000001134 | Uxt | 0.98 | 2.56E-03 |
| ENSMUSG00000059305 | Vpreb1 | 0.98 | 2.15E-02 |
| ENSMUSG00000040274 | Cdk6 | 0.98 | 1.14E-02 |
| ENSMUSG00000059182 | Skap2 | 0.96 | 5.14E-06 |
| ENSMUSG00000032067 | Pts | 0.94 | 2.13E-02 |
| ENSMUSG00000022747 | St3gal6 | 0.94 | 2.79E-02 |
| ENSMUSG00000029911 | Ssbp1 | 0.93 | 1.77E-02 |
| ENSMUSG00000031613 | Hpgd | 0.93 | 3.10E-02 |
| ENSMUSG00000074486 | Bglap2 | 0.93 | 8.84E-04 |
| ENSMUSG00000021024 | Psma6 | 0.93 | 6.37E-03 |
| ENSMUSG00000004446 | Bid | 0.92 | 3.37E-02 |
| ENSMUSG00000024529 | Lox | 0.89 | 1.79E-02 |
| ENSMUSG00000050493 | Fam167b | 0.87 | 3.96E-02 |
| ENSMUSG00000009630 | Ppp2cb | 0.87 | 3.33E-03 |
| ENSMUSG00000031962 | Cdh15 | 0.86 | 5.00E-02 |
| ENSMUSG00000037601 | Nme1 | 0.86 | 1.77E-02 |
| ENSMUSG00000061353 | Cxcl12 | 0.86 | 1.71E-03 |
| ENSMUSG00000032556 | Bfsp2 | 0.85 | 4.33E-02 |
| ENSMUSG00000063480 | Snu13 | 0.84 | 1.22E-02 |
| ENSMUSG00000019777 | Hdac2 | 0.84 | 4.23E-02 |
| ENSMUSG00000004933 | Matk | 0.83 | 3.79E-02 |
| ENSMUSG00000026377 | Nifk | 0.83 | 4.61E-02 |
| ENSMUSG00000032412 | Atp1b3 | 0.83 | 1.94E-07 |
| ENSMUSG00000047557 | Lxn | 0.83 | 9.76E-03 |
| ENSMUSG00000029161 | Cgref1 | 0.82 | 2.59E-02 |
| ENSMUSG00000030513 | Pcsk6 | 0.82 | 2.10E-02 |
| ENSMUSG00000032370 | Lactb | 0.82 | 4.78E-02 |
| ENSMUSG00000032766 | Gng11 | 0.81 | 1.20E-02 |
| ENSMUSG00000030423 | Pop4 | 0.81 | 4.44E-02 |
| ENSMUSG00000000420 | Galnt1 | 0.80 | 3.43E-02 |
| ENSMUSG00000028647 | Mycbp | 0.78 | 2.45E-02 |
| ENSMUSG00000027207 | Galk2 | 0.77 | 2.46E-02 |
| ENSMUSG00000025747 | Tyms | 0.76 | 2.77E-02 |
| ENSMUSG00000023791 | Pigx | 0.76 | 4.01E-02 |
| ENSMUSG00000032207 | Lipc | 0.76 | 3.60E-02 |
| ENSMUSG00000039007 | Cpq | 0.76 | 1.83E-02 |
| ENSMUSG00000028271 | Gtf2b | 0.75 | 8.94E-03 |
| ENSMUSG00000025915 | Sgk3 | 0.75 | 4.67E-02 |
| ENSMUSG00000020869 | Lrrc59 | 0.74 | 4.64E-02 |
| ENSMUSG00000020357 | Flt4 | 0.74 | 3.51E-02 |
| ENSMUSG00000034544 | Rsrc1 | 0.74 | 2.97E-02 |
| ENSMUSG00000037852 | Cpe | 0.73 | 1.42E-02 |
| ENSMUSG00000002014 | Ssr4 | 0.73 | 3.35E-05 |
| ENSMUSG00000018189 | Uchl5 | 0.72 | 1.70E-02 |
| ENSMUSG00000024018 | Ccdc167 | 0.72 | 3.84E-02 |
| ENSMUSG00000031980 | Agt | 0.72 | 3.33E-02 |
| ENSMUSG00000027879 | Sec22b | 0.70 | 2.77E-02 |
| ENSMUSG00000105814 | Mir703 | 0.70 | 2.16E-02 |
| ENSMUSG00000028364 | Tnc | 0.70 | 2.55E-02 |
| ENSMUSG00000044627 | Swi5 | 0.70 | 5.24E-05 |
| ENSMUSG00000023367 | Tmem176a | 0.70 | 5.19E-03 |
| ENSMUSG00000028076 | Cd1d1 | 0.70 | 2.24E-02 |
| ENSMUSG00000032757 | Bet1 | 0.69 | 3.80E-02 |
| ENSMUSG00000006273 | Atp6v1b2 | 0.68 | 1.67E-02 |
| ENSMUSG00000024067 | Dpy30 | 0.67 | 1.35E-02 |
| ENSMUSG00000031683 | Lsm6 | 0.67 | 2.51E-02 |
| ENSMUSG00000016319 | Slc25a5 | 0.67 | 8.73E-04 |
| ENSMUSG00000028494 | Plin2 | 0.67 | 4.60E-02 |
| ENSMUSG00000020689 | Itgb3 | 0.66 | 2.22E-03 |
| ENSMUSG00000058715 | Fcer1g | 0.66 | 1.66E-02 |
| ENSMUSG00000020328 | Nudcd2 | 0.66 | 2.55E-02 |
| ENSMUSG00000020706 | Ftsj3 | 0.64 | 2.08E-02 |
| ENSMUSG00000053604 | Rpia | 0.63 | 3.61E-02 |
| ENSMUSG00000033845 | Mrpl15 | 0.63 | 4.64E-02 |
| ENSMUSG00000031954 | Cfdp1 | 0.62 | 1.00E-02 |
| ENSMUSG00000040549 | Ckap5 | 0.62 | 4.01E-02 |
| ENSMUSG00000001348 | Acp5 | 0.62 | 4.09E-02 |
| ENSMUSG00000026126 | Ptpn18 | 0.61 | 1.57E-02 |
| ENSMUSG00000061787 | Rps17 | 0.61 | 5.33E-05 |
| ENSMUSG00000022205 | Sub1 | 0.61 | 2.76E-02 |
| ENSMUSG00000058351 | Smim4 | 0.61 | 1.83E-02 |
| ENSMUSG00000028936 | Rpl22 | 0.60 | 3.88E-02 |
| ENSMUSG00000022174 | Dad1 | 0.59 | 1.40E-02 |
| ENSMUSG00000030579 | Tyrobp | 0.59 | 1.35E-02 |
| ENSMUSG00000029071 | Dvl1 | -0.59 | 3.76E-02 |
| ENSMUSG00000021756 | Il6st | -0.59 | 8.73E-04 |
| ENSMUSG00000023830 | Igf2r | -0.60 | 7.55E-03 |
| ENSMUSG00000066621 | Tecpr1 | -0.60 | 3.29E-02 |
| ENSMUSG00000032479 | Map4 | -0.61 | 5.87E-03 |
| ENSMUSG00000024610 | Cd74 | -0.62 | 3.43E-03 |
| ENSMUSG00000025579 | Gaa | -0.62 | 1.40E-02 |
| ENSMUSG00000042608 | Stk40 | -0.62 | 9.94E-03 |
| ENSMUSG00000027602 | Map1lc3a | -0.62 | 4.98E-02 |
| ENSMUSG00000061410 | Zcchc14 | -0.62 | 3.60E-02 |
| ENSMUSG00000005514 | Por | -0.63 | 5.07E-03 |
| ENSMUSG00000020743 | Mif4gd | -0.63 | 3.20E-02 |
| ENSMUSG00000023055 | Calcoco1 | -0.64 | 2.96E-02 |
| ENSMUSG00000037992 | Rara | -0.65 | 4.99E-02 |
| ENSMUSG00000018574 | Acadvl | -0.65 | 2.91E-02 |
| ENSMUSG00000026483 | Fam129a | -0.65 | 3.07E-02 |
| ENSMUSG00000000278 | Scpep1 | -0.65 | 1.55E-02 |
| ENSMUSG00000050212 | Eva1b | -0.65 | 2.78E-02 |
| ENSMUSG00000034135 | Sik3 | -0.65 | 5.93E-03 |
| ENSMUSG00000038615 | Nfe2l1 | -0.65 | 4.44E-04 |
| ENSMUSG00000032737 | Inppl1 | -0.65 | 4.32E-02 |
| ENSMUSG00000008575 | Nfib | -0.65 | 3.06E-02 |
| ENSMUSG00000027288 | Zfp106 | -0.65 | 8.53E-04 |
| ENSMUSG00000001098 | Kctd10 | -0.65 | 1.48E-03 |
| ENSMUSG00000030527 | Crtc3 | -0.66 | 2.70E-02 |
| ENSMUSG00000028854 | Slc9a1 | -0.66 | 7.95E-03 |
| ENSMUSG00000053617 | Sh3pxd2a | -0.66 | 2.12E-02 |
| ENSMUSG00000015305 | Sash1 | -0.67 | 2.74E-02 |
| ENSMUSG00000045252 | Zfp574 | -0.67 | 4.90E-02 |
| ENSMUSG00000042903 | Foxo4 | -0.67 | 2.24E-02 |
| ENSMUSG00000036966 | Spryd3 | -0.67 | 2.70E-02 |
| ENSMUSG00000026814 | Eng | -0.68 | 8.51E-04 |
| ENSMUSG00000028959 | Fastk | -0.68 | 1.66E-02 |
| ENSMUSG00000025571 | Tnrc6c | -0.68 | 1.65E-03 |
| ENSMUSG00000021838 | Samd4 | -0.68 | 1.96E-02 |
| ENSMUSG00000022620 | Arsa | -0.68 | 1.58E-02 |
| ENSMUSG00000031451 | Gas6 | -0.68 | 1.53E-03 |
| ENSMUSG00000020176 | Grb10 | -0.68 | 9.01E-03 |
| ENSMUSG00000055538 | Zcchc24 | -0.68 | 1.57E-02 |
| ENSMUSG00000024921 | Smarca2 | -0.68 | 3.36E-02 |
| ENSMUSG00000032855 | Pkd1 | -0.69 | 2.22E-02 |
| ENSMUSG00000085795 | Zfp703 | -0.69 | 5.38E-03 |
| ENSMUSG00000023022 | Lima1 | -0.69 | 2.05E-02 |
| ENSMUSG00000028756 | Pink1 | -0.69 | 5.19E-03 |
| ENSMUSG00000055799 | Tcf7l1 | -0.69 | 2.27E-02 |
| ENSMUSG00000030201 | Lrp6 | -0.70 | 3.82E-02 |
| ENSMUSG00000055053 | Nfic | -0.70 | 9.46E-03 |
| ENSMUSG00000039952 | Dag1 | -0.70 | 1.75E-03 |
| ENSMUSG00000012296 | Tjap1 | -0.70 | 6.10E-03 |
| ENSMUSG00000063382 | Bcl9l | -0.71 | 2.36E-02 |
| ENSMUSG00000030987 | Stim1 | -0.71 | 5.27E-04 |
| ENSMUSG00000067787 | Blcap | -0.71 | 3.42E-02 |
| ENSMUSG00000000957 | Mmp14 | -0.71 | 4.91E-02 |
| ENSMUSG00000024193 | Phf1 | -0.72 | 2.27E-02 |
| ENSMUSG00000027864 | Ptgfrn | -0.72 | 4.71E-02 |
| ENSMUSG00000003316 | Glg1 | -0.72 | 3.44E-03 |
| ENSMUSG00000004263 | Atn1 | -0.72 | 3.40E-02 |
| ENSMUSG00000042507 | Elmsan1 | -0.73 | 2.70E-02 |
| ENSMUSG00000030782 | Tgfb1i1 | -0.74 | 8.26E-03 |
| ENSMUSG00000020520 | Galnt10 | -0.74 | 1.32E-03 |
| ENSMUSG00000024299 | Adamts10 | -0.75 | 1.55E-02 |
| ENSMUSG00000097328 | Tnfsf12 | -0.75 | 4.80E-02 |
| ENSMUSG00000037206 | Islr | -0.76 | 2.65E-03 |
| ENSMUSG00000037060 | Cavin3 | -0.76 | 3.26E-03 |
| ENSMUSG00000029432 | Nipsnap2 | -0.76 | 1.57E-02 |
| ENSMUSG00000051343 | Rab11fip5 | -0.77 | 2.89E-02 |
| ENSMUSG00000020821 | Kif1c | -0.77 | 2.99E-02 |
| ENSMUSG00000029392 | Rilpl1 | -0.78 | 4.46E-02 |
| ENSMUSG00000040732 | Erg | -0.78 | 1.20E-02 |
| ENSMUSG00000063160 | Numbl | -0.78 | 3.49E-02 |
| ENSMUSG00000063077 | Kif1b | -0.78 | 3.23E-02 |
| ENSMUSG00000033295 | Ptprf | -0.78 | 5.84E-03 |
| ENSMUSG00000031990 | Jam3 | -0.78 | 2.42E-02 |
| ENSMUSG00000096188 | Cmtm4 | -0.79 | 1.69E-02 |
| ENSMUSG00000004098 | Col5a3 | -0.79 | 7.60E-03 |
| ENSMUSG00000015501 | Hivep2 | -0.79 | 4.23E-02 |
| ENSMUSG00000020486 | Septin4 | -0.80 | 9.24E-04 |
| ENSMUSG00000012017 | Scarf2 | -0.80 | 1.53E-02 |
| ENSMUSG00000038539 | Atf5 | -0.80 | 1.23E-03 |
| ENSMUSG00000036686 | Cc2d1a | -0.80 | 4.24E-02 |
| ENSMUSG00000018428 | Akap1 | -0.80 | 2.74E-02 |
| ENSMUSG00000026510 | Trp53bp2 | -0.81 | 4.00E-02 |
| ENSMUSG00000029050 | Ski | -0.81 | 1.49E-03 |
| ENSMUSG00000020154 | Ptprb | -0.81 | 9.46E-03 |
| ENSMUSG00000069833 | Ahnak | -0.81 | 1.40E-03 |
| ENSMUSG00000004044 | Cavin1 | -0.82 | 1.05E-03 |
| ENSMUSG00000020439 | Smtn | -0.82 | 6.07E-03 |
| ENSMUSG00000032243 | Itga11 | -0.82 | 2.75E-02 |
| ENSMUSG00000027253 | Lrp4 | -0.82 | 1.12E-02 |
| ENSMUSG00000052911 | Lamb2 | -0.82 | 1.56E-02 |
| ENSMUSG00000020674 | Pxdn | -0.82 | 4.62E-02 |
| ENSMUSG00000043903 | Zfp469 | -0.82 | 1.89E-02 |
| ENSMUSG00000014329 | Bicc1 | -0.83 | 2.01E-02 |
| ENSMUSG00000058254 | Tspan7 | -0.83 | 9.24E-04 |
| ENSMUSG00000030281 | Il17rc | -0.83 | 3.85E-02 |
| ENSMUSG00000020044 | Timp3 | -0.84 | 6.93E-04 |
| ENSMUSG00000036989 | Trim3 | -0.85 | 3.35E-02 |
| ENSMUSG00000026072 | Il1r1 | -0.85 | 1.67E-02 |
| ENSMUSG00000031840 | Rab3a | -0.85 | 2.82E-02 |
| ENSMUSG00000032816 | Igdcc4 | -0.85 | 4.53E-02 |
| ENSMUSG00000024962 | Vegfb | -0.86 | 3.67E-03 |
| ENSMUSG00000020387 | Jade2 | -0.86 | 3.82E-02 |
| ENSMUSG00000006301 | Tmbim1 | -0.86 | 2.51E-04 |
| ENSMUSG00000026490 | Cdc42bpa | -0.87 | 1.54E-04 |
| ENSMUSG00000026131 | Dst | -0.87 | 8.58E-04 |
| ENSMUSG00000032334 | Loxl1 | -0.87 | 4.50E-03 |
| ENSMUSG00000037712 | Fermt2 | -0.87 | 1.29E-02 |
| ENSMUSG00000060572 | Mfap2 | -0.88 | 8.63E-03 |
| ENSMUSG00000083282 | Ctsf | -0.88 | 1.24E-05 |
| ENSMUSG00000027551 | Zfp64 | -0.88 | 2.53E-02 |
| ENSMUSG00000013419 | Zfp651 | -0.88 | 1.97E-02 |
| ENSMUSG00000029060 | Mib2 | -0.88 | 4.16E-02 |
| ENSMUSG00000030259 | Rassf8 | -0.88 | 6.20E-03 |
| ENSMUSG00000039542 | Ncam1 | -0.88 | 9.54E-06 |
| ENSMUSG00000020427 | Igfbp3 | -0.89 | 8.94E-03 |
| ENSMUSG00000026930 | Gpsm1 | -0.89 | 5.20E-03 |
| ENSMUSG00000023249 | Parp3 | -0.89 | 7.24E-04 |
| ENSMUSG00000070565 | Rasal2 | -0.89 | 3.31E-02 |
| ENSMUSG00000022708 | Zbtb20 | -0.89 | 2.06E-02 |
| ENSMUSG00000023224 | Serping1 | -0.90 | 3.09E-05 |
| ENSMUSG00000028063 | Lmna | -0.91 | 3.03E-05 |
| ENSMUSG00000031595 | Pdgfrl | -0.91 | 2.29E-03 |
| ENSMUSG00000019467 | Arhgef25 | -0.92 | 5.55E-03 |
| ENSMUSG00000040488 | Ltbp4 | -0.92 | 1.97E-02 |
| ENSMUSG00000024620 | Pdgfrb | -0.93 | 1.48E-03 |
| ENSMUSG00000033420 | Antxr1 | -0.93 | 1.10E-03 |
| ENSMUSG00000018217 | Pmp22 | -0.93 | 2.37E-02 |
| ENSMUSG00000022265 | Ank | -0.94 | 8.85E-08 |
| ENSMUSG00000051790 | Nlgn2 | -0.95 | 9.76E-03 |
| ENSMUSG00000040249 | Lrp1 | -0.96 | 5.54E-07 |
| ENSMUSG00000032289 | Thsd4 | -0.96 | 9.44E-03 |
| ENSMUSG00000089715 | Cbx6 | -0.97 | 5.00E-10 |
| ENSMUSG00000058756 | Thra | -0.97 | 2.49E-07 |
| ENSMUSG00000030218 | Mgp | -0.97 | 4.89E-02 |
| ENSMUSG00000017466 | Timp2 | -0.98 | 7.23E-06 |
| ENSMUSG00000018861 | Fdxr | -0.99 | 5.93E-03 |
| ENSMUSG00000039953 | Clstn1 | -0.99 | 1.69E-05 |
| ENSMUSG00000038248 | Sobp | -0.99 | 2.88E-02 |
| ENSMUSG00000053310 | Nrgn | -0.99 | 8.01E-03 |
| ENSMUSG00000029675 | Eln | -0.99 | 1.04E-02 |
| ENSMUSG00000020142 | Slc1a4 | -1.00 | 2.76E-02 |
| ENSMUSG00000055320 | Tead1 | -1.00 | 3.96E-03 |
| ENSMUSG00000022562 | Oplah | -1.01 | 4.01E-02 |
| ENSMUSG00000055172 | C1ra | -1.01 | 3.26E-03 |
| ENSMUSG00000002249 | Tead3 | -1.02 | 3.24E-02 |
| ENSMUSG00000020393 | Kremen1 | -1.03 | 1.33E-04 |
| ENSMUSG00000024168 | Tmem204 | -1.03 | 5.05E-03 |
| ENSMUSG00000032340 | Neo1 | -1.03 | 1.10E-05 |
| ENSMUSG00000032733 | Snx33 | -1.03 | 1.55E-02 |
| ENSMUSG00000004562 | Arhgef40 | -1.04 | 3.10E-04 |
| ENSMUSG00000024420 | Zfp521 | -1.04 | 4.75E-02 |
| ENSMUSG00000028207 | Asph | -1.04 | 1.97E-02 |
| ENSMUSG00000022505 | Emp2 | -1.05 | 2.23E-03 |
| ENSMUSG00000031431 | Tsc22d3 | -1.06 | 5.24E-05 |
| ENSMUSG00000033209 | Ttc28 | -1.06 | 7.34E-04 |
| ENSMUSG00000041559 | Fmod | -1.06 | 1.66E-02 |
| ENSMUSG00000051367 | Six1 | -1.06 | 2.70E-02 |
| ENSMUSG00000002059 | Rab34 | -1.07 | 2.27E-04 |
| ENSMUSG00000001288 | Rarg | -1.08 | 1.30E-04 |
| ENSMUSG00000024247 | Pkdcc | -1.08 | 1.30E-04 |
| ENSMUSG00000023034 | Nr4a1 | -1.09 | 2.31E-02 |
| ENSMUSG00000026185 | Igfbp5 | -1.09 | 5.00E-10 |
| ENSMUSG00000028199 | Cryz | -1.09 | 3.70E-02 |
| ENSMUSG00000039062 | Anpep | -1.09 | 3.60E-02 |
| ENSMUSG00000030255 | Sspn | -1.10 | 2.84E-02 |
| ENSMUSG00000021253 | Tgfb3 | -1.11 | 1.54E-05 |
| ENSMUSG00000042821 | Snai1 | -1.11 | 4.20E-04 |
| ENSMUSG00000066705 | Fxyd6 | -1.11 | 3.82E-03 |
| ENSMUSG00000019194 | Scn1b | -1.12 | 2.67E-02 |
| ENSMUSG00000022223 | Sdr39u1 | -1.12 | 2.71E-02 |
| ENSMUSG00000031993 | Snx19 | -1.12 | 1.70E-02 |
| ENSMUSG00000034751 | Mast4 | -1.12 | 4.47E-04 |
| ENSMUSG00000039976 | Tbc1d16 | -1.12 | 4.35E-02 |
| ENSMUSG00000052934 | Fbxo31 | -1.12 | 5.05E-03 |
| ENSMUSG00000063605 | Ccdc102a | -1.12 | 2.09E-02 |
| ENSMUSG00000002799 | Jag2 | -1.13 | 4.14E-02 |
| ENSMUSG00000022893 | Adamts1 | -1.13 | 1.17E-02 |
| ENSMUSG00000026586 | Prrx1 | -1.13 | 1.42E-03 |
| ENSMUSG00000007783 | Cpt1c | -1.14 | 3.23E-02 |
| ENSMUSG00000017754 | Pltp | -1.14 | 7.67E-04 |
| ENSMUSG00000018566 | Slc2a4 | -1.14 | 2.26E-02 |
| ENSMUSG00000025355 | Mmp19 | -1.14 | 2.70E-02 |
| ENSMUSG00000038668 | Lpar1 | -1.14 | 3.31E-06 |
| ENSMUSG00000021268 | Meg3 | -1.15 | 5.00E-02 |
| ENSMUSG00000034161 | Scx | -1.15 | 3.23E-03 |
| ENSMUSG00000036278 | Macrod1 | -1.15 | 3.29E-02 |
| ENSMUSG00000053863 | Mepe | -1.15 | 4.20E-04 |
| ENSMUSG00000022565 | Plec | -1.16 | 6.10E-05 |
| ENSMUSG00000023805 | Synj2 | -1.16 | 3.75E-02 |
| ENSMUSG00000031633 | Slc25a4 | -1.16 | 1.72E-03 |
| ENSMUSG00000051373 | Plpp7 | -1.16 | 3.42E-02 |
| ENSMUSG00000039239 | Tgfb2 | -1.17 | 2.38E-03 |
| ENSMUSG00000048126 | Col6a3 | -1.17 | 4.06E-05 |
| ENSMUSG00000026825 | Dnm1 | -1.18 | 4.57E-03 |
| ENSMUSG00000040280 | Ndufa4l2 | -1.18 | 3.11E-02 |
| ENSMUSG00000027546 | Atp9a | -1.19 | 4.74E-04 |
| ENSMUSG00000046727 | Cystm1 | -1.20 | 2.47E-02 |
| ENSMUSG00000071064 | Zfp827 | -1.20 | 2.84E-02 |
| ENSMUSG00000021767 | Kat6b | -1.21 | 1.39E-04 |
| ENSMUSG00000030323 | Ift122 | -1.21 | 5.20E-03 |
| ENSMUSG00000031748 | Gnao1 | -1.21 | 2.52E-02 |
| ENSMUSG00000038967 | Pdk2 | -1.21 | 1.08E-02 |
| ENSMUSG00000026817 | Ak1 | -1.22 | 3.50E-03 |
| ENSMUSG00000000538 | Tom1l2 | -1.23 | 3.24E-05 |
| ENSMUSG00000039474 | Wfs1 | -1.23 | 2.35E-03 |
| ENSMUSG00000042485 | Mustn1 | -1.23 | 7.98E-03 |
| ENSMUSG00000027318 | Adam33 | -1.24 | 3.56E-03 |
| ENSMUSG00000059824 | Dbp | -1.24 | 1.11E-02 |
| ENSMUSG00000031486 | Adgra2 | -1.26 | 1.15E-05 |
| ENSMUSG00000033102 | Cdc14b | -1.26 | 2.62E-02 |
| ENSMUSG00000041308 | Sntb2 | -1.26 | 9.73E-03 |
| ENSMUSG00000052688 | Rab7b | -1.26 | 4.21E-03 |
| ENSMUSG00000005397 | Nid1 | -1.27 | 1.02E-02 |
| ENSMUSG00000078794 | Dact3 | -1.27 | 5.88E-03 |
| ENSMUSG00000021559 | Dapk1 | -1.28 | 3.52E-03 |
| ENSMUSG00000038305 | Spats2l | -1.28 | 1.67E-02 |
| ENSMUSG00000039081 | Zfp503 | -1.28 | 8.01E-03 |
| ENSMUSG00000000142 | Axin2 | -1.29 | 2.47E-02 |
| ENSMUSG00000001911 | Nfix | -1.29 | 2.89E-08 |
| ENSMUSG00000022836 | Mylk | -1.29 | 5.12E-08 |
| ENSMUSG00000031596 | Slc7a2 | -1.29 | 3.03E-02 |
| ENSMUSG00000039914 | Coq10a | -1.29 | 2.19E-02 |
| ENSMUSG00000038170 | Pde4dip | -1.30 | 3.43E-03 |
| ENSMUSG00000024617 | Camk2a | -1.31 | 3.27E-03 |
| ENSMUSG00000026676 | Ccdc3 | -1.31 | 1.76E-03 |
| ENSMUSG00000022421 | Nptxr | -1.32 | 3.23E-02 |
| ENSMUSG00000031616 | Ednra | -1.32 | 3.76E-02 |
| ENSMUSG00000017897 | Eya2 | -1.33 | 2.91E-03 |
| ENSMUSG00000032238 | Rora | -1.34 | 1.65E-03 |
| ENSMUSG00000056481 | Cd248 | -1.34 | 9.46E-03 |
| ENSMUSG00000002980 | Bcam | -1.35 | 2.17E-03 |
| ENSMUSG00000030772 | Dkk3 | -1.35 | 2.96E-06 |
| ENSMUSG00000027340 | Slc23a2 | -1.36 | 2.98E-04 |
| ENSMUSG00000040287 | Stac3 | -1.36 | 4.78E-02 |
| ENSMUSG00000020241 | Col6a2 | -1.37 | 1.78E-06 |
| ENSMUSG00000027408 | Cpxm1 | -1.38 | 1.22E-04 |
| ENSMUSG00000032085 | Tagln | -1.38 | 3.67E-04 |
| ENSMUSG00000034839 | Larp6 | -1.39 | 5.88E-03 |
| ENSMUSG00000045312 | Lhfpl2 | -1.39 | 2.48E-03 |
| ENSMUSG00000003070 | Efna2 | -1.40 | 1.20E-02 |
| ENSMUSG00000014846 | Tppp3 | -1.42 | 1.07E-03 |
| ENSMUSG00000022594 | Lynx1 | -1.42 | 6.27E-04 |
| ENSMUSG00000040998 | Npnt | -1.42 | 1.74E-04 |
| ENSMUSG00000028047 | Thbs3 | -1.44 | 1.03E-03 |
| ENSMUSG00000044548 | Dact1 | -1.44 | 3.31E-02 |
| ENSMUSG00000041548 | Hspb8 | -1.45 | 5.44E-03 |
| ENSMUSG00000064080 | Fbln2 | -1.45 | 1.82E-06 |
| ENSMUSG00000038239 | Hrc | -1.46 | 2.80E-02 |
| ENSMUSG00000038738 | Shank1 | -1.46 | 4.84E-03 |
| ENSMUSG00000034118 | Tpst1 | -1.47 | 6.53E-06 |
| ENSMUSG00000032060 | Cryab | -1.48 | 3.67E-04 |
| ENSMUSG00000033585 | Ndn | -1.48 | 7.82E-04 |
| ENSMUSG00000027750 | Postn | -1.49 | 4.26E-03 |
| ENSMUSG00000033863 | Klf9 | -1.50 | 0.00E+00 |
| ENSMUSG00000020814 | Mxra7 | -1.51 | 3.11E-06 |
| ENSMUSG00000037621 | Atoh8 | -1.51 | 1.06E-05 |
| ENSMUSG00000044006 | Cilp2 | -1.51 | 1.25E-02 |
| ENSMUSG00000072812 | Ahnak2 | -1.51 | 5.20E-03 |
| ENSMUSG00000013921 | Clip3 | -1.52 | 6.40E-05 |
| ENSMUSG00000023885 | Thbs2 | -1.52 | 1.94E-07 |
| ENSMUSG00000039376 | Synpo2l | -1.52 | 4.07E-02 |
| ENSMUSG00000001119 | Col6a1 | -1.55 | 1.16E-08 |
| ENSMUSG00000028464 | Tpm2 | -1.55 | 2.99E-02 |
| ENSMUSG00000028909 | Ptpru | -1.55 | 8.37E-03 |
| ENSMUSG00000020810 | Cygb | -1.57 | 6.58E-07 |
| ENSMUSG00000006205 | Htra1 | -1.58 | 1.34E-08 |
| ENSMUSG00000021200 | Asb2 | -1.58 | 3.85E-03 |
| ENSMUSG00000020388 | Pdlim4 | -1.60 | 1.50E-08 |
| ENSMUSG00000023411 | Nfatc4 | -1.60 | 5.33E-05 |
| ENSMUSG00000045667 | Smtnl2 | -1.60 | 1.14E-02 |
| ENSMUSG00000020866 | Cacna1g | -1.61 | 2.70E-09 |
| ENSMUSG00000029673 | Auts2 | -1.61 | 2.98E-06 |
| ENSMUSG00000056900 | Usp13 | -1.61 | 2.87E-02 |
| ENSMUSG00000027544 | Nfatc2 | -1.62 | 4.21E-03 |
| ENSMUSG00000062563 | Cys1 | -1.62 | 7.98E-03 |
| ENSMUSG00000004105 | Angptl2 | -1.63 | 2.18E-07 |
| ENSMUSG00000004558 | Ndrg2 | -1.63 | 1.14E-02 |
| ENSMUSG00000038370 | Pcp4l1 | -1.64 | 2.68E-05 |
| ENSMUSG00000039103 | Nexn | -1.64 | 3.06E-02 |
| ENSMUSG00000024076 | Vit | -1.65 | 7.72E-03 |
| ENSMUSG00000057604 | Lmcd1 | -1.65 | 3.02E-04 |
| ENSMUSG00000018339 | Gpx3 | -1.66 | 9.90E-07 |
| ENSMUSG00000028664 | Ephb2 | -1.66 | 7.67E-04 |
| ENSMUSG00000000031 | H19 | -1.67 | 1.24E-03 |
| ENSMUSG00000005958 | Ephb3 | -1.67 | 1.57E-04 |
| ENSMUSG00000022340 | Sybu | -1.68 | 6.20E-03 |
| ENSMUSG00000020793 | Galr2 | -1.69 | 2.75E-03 |
| ENSMUSG00000032010 | Usp2 | -1.69 | 3.55E-03 |
| ENSMUSG00000035799 | Twist1 | -1.69 | 1.00E-10 |
| ENSMUSG00000001665 | Gstt3 | -1.70 | 1.56E-02 |
| ENSMUSG00000097324 | Carmn | -1.70 | 4.78E-02 |
| ENSMUSG00000019899 | Lama2 | -1.71 | 4.45E-03 |
| ENSMUSG00000031791 | Tmem38a | -1.71 | 3.79E-02 |
| ENSMUSG00000039323 | Igfbp2 | -1.71 | 4.56E-04 |
| ENSMUSG00000038233 | Gask1a | -1.72 | 5.48E-03 |
| ENSMUSG00000020473 | Aebp1 | -1.73 | 3.22E-07 |
| ENSMUSG00000029838 | Ptn | -1.73 | 5.20E-09 |
| ENSMUSG00000035783 | Acta2 | -1.73 | 2.07E-07 |
| ENSMUSG00000038204 | Asb10 | -1.73 | 1.38E-02 |
| ENSMUSG00000042686 | Jph1 | -1.73 | 2.84E-02 |
| ENSMUSG00000002020 | Ltbp2 | -1.74 | 1.31E-03 |
| ENSMUSG00000015850 | Adamtsl4 | -1.74 | 1.57E-02 |
| ENSMUSG00000028773 | Fabp3 | -1.74 | 1.96E-02 |
| ENSMUSG00000017300 | Tnnc2 | -1.75 | 4.14E-02 |
| ENSMUSG00000028128 | F3 | -1.75 | 1.67E-02 |
| ENSMUSG00000031250 | Tnmd | -1.75 | 2.76E-03 |
| ENSMUSG00000031778 | Cx3cl1 | -1.75 | 1.83E-02 |
| ENSMUSG00000063838 | Cdc42ep5 | -1.75 | 6.81E-06 |
| ENSMUSG00000004951 | Hspb1 | -1.76 | 1.28E-03 |
| ENSMUSG00000026208 | Des | -1.76 | 3.66E-02 |
| ENSMUSG00000027412 | Lpin3 | -1.77 | 1.70E-02 |
| ENSMUSG00000033965 | Slc16a2 | -1.77 | 3.79E-03 |
| ENSMUSG00000090799 | Klhl33 | -1.77 | 4.46E-03 |
| ENSMUSG00000078915 | Hsp25-ps1 | -1.78 | 3.80E-02 |
| ENSMUSG00000001604 | Tcea3 | -1.79 | 1.45E-04 |
| ENSMUSG00000021062 | Rab15 | -1.80 | 3.49E-02 |
| ENSMUSG00000026950 | Neb | -1.81 | 4.60E-02 |
| ENSMUSG00000033576 | Apol6 | -1.81 | 5.07E-03 |
| ENSMUSG00000025407 | Gli1 | -1.82 | 5.33E-05 |
| ENSMUSG00000035279 | Ssc5d | -1.82 | 2.29E-06 |
| ENSMUSG00000051747 | Ttn | -1.82 | 3.49E-02 |
| ENSMUSG00000028116 | Myoz2 | -1.86 | 4.79E-02 |
| ENSMUSG00000038872 | Zfhx3 | -1.88 | 1.40E-05 |
| ENSMUSG00000040694 | Apobec2 | -1.88 | 4.14E-02 |
| ENSMUSG00000079588 | Tmem182 | -1.88 | 1.67E-02 |
| ENSMUSG00000028601 | Echdc2 | -1.89 | 1.79E-05 |
| ENSMUSG00000026235 | Epha4 | -1.90 | 7.72E-05 |
| ENSMUSG00000030852 | Tacc2 | -1.90 | 1.02E-06 |
| ENSMUSG00000027257 | Pacsin3 | -1.91 | 1.33E-05 |
| ENSMUSG00000006800 | Sulf2 | -1.92 | 1.00E-10 |
| ENSMUSG00000009281 | Rarres2 | -1.92 | 1.50E-08 |
| ENSMUSG00000031636 | Pdlim3 | -1.92 | 5.56E-03 |
| ENSMUSG00000043822 | Adamtsl5 | -1.93 | 1.74E-04 |
| ENSMUSG00000079243 | Xirp1 | -1.93 | 4.16E-02 |
| ENSMUSG00000006369 | Fbln1 | -1.94 | 4.56E-06 |
| ENSMUSG00000046818 | Ddit4l | -1.95 | 1.71E-02 |
| ENSMUSG00000050737 | Ptges | -1.95 | 2.53E-02 |
| ENSMUSG00000022519 | Srl | -1.96 | 5.55E-03 |
| ENSMUSG00000007097 | Atp1a2 | -1.97 | 1.66E-02 |
| ENSMUSG00000025488 | Cox8b | -1.97 | 4.49E-02 |
| ENSMUSG00000021464 | Ror2 | -1.98 | 2.18E-07 |
| ENSMUSG00000039883 | Lrrc17 | -1.98 | 7.31E-08 |
| ENSMUSG00000062077 | Trim54 | -1.98 | 2.14E-02 |
| ENSMUSG00000062694 | Cav3 | -1.98 | 2.02E-03 |
| ENSMUSG00000044674 | Fzd1 | -1.99 | 8.85E-08 |
| ENSMUSG00000040690 | Col16a1 | -2.01 | 0.00E+00 |
| ENSMUSG00000056569 | Mpz | -2.01 | 2.70E-02 |
| ENSMUSG00000007279 | Scube2 | -2.02 | 8.31E-04 |
| ENSMUSG00000054034 | Tceal5 | -2.03 | 1.04E-02 |
| ENSMUSG00000030996 | Art1 | -2.04 | 4.80E-03 |
| ENSMUSG00000031312 | Itgb1bp2 | -2.04 | 1.25E-02 |
| ENSMUSG00000020173 | Cobl | -2.06 | 4.84E-02 |
| ENSMUSG00000048416 | Mlf1 | -2.06 | 2.91E-02 |
| ENSMUSG00000000126 | Wnt9a | -2.08 | 5.92E-03 |
| ENSMUSG00000030785 | Cox6a2 | -2.09 | 1.20E-02 |
| ENSMUSG00000046480 | Scn4b | -2.09 | 5.15E-03 |
| ENSMUSG00000068697 | Myoz1 | -2.09 | 2.53E-02 |
| ENSMUSG00000097993 | Ptprv | -2.09 | 1.75E-06 |
| ENSMUSG00000020105 | Lrig3 | -2.10 | 1.08E-04 |
| ENSMUSG00000022296 | Baalc | -2.10 | 1.75E-03 |
| ENSMUSG00000022371 | Col14a1 | -2.10 | 4.42E-05 |
| ENSMUSG00000022817 | Itgb5 | -2.10 | 0.00E+00 |
| ENSMUSG00000038521 | C1s1 | -2.11 | 1.23E-05 |
| ENSMUSG00000068699 | Flnc | -2.11 | 5.45E-05 |
| ENSMUSG00000016262 | Sertad4 | -2.13 | 2.96E-06 |
| ENSMUSG00000020889 | Nr1d1 | -2.14 | 1.94E-08 |
| ENSMUSG00000030730 | Atp2a1 | -2.15 | 1.46E-02 |
| ENSMUSG00000021798 | Ldb3 | -2.16 | 1.44E-02 |
| ENSMUSG00000024059 | Clip4 | -2.16 | 2.36E-05 |
| ENSMUSG00000032011 | Thy1 | -2.17 | 0.00E+00 |
| ENSMUSG00000005373 | Mlxipl | -2.18 | 3.06E-02 |
| ENSMUSG00000020061 | Mybpc1 | -2.18 | 1.57E-02 |
| ENSMUSG00000020722 | Cacng1 | -2.18 | 5.88E-03 |
| ENSMUSG00000023153 | Tmem52 | -2.18 | 3.40E-02 |
| ENSMUSG00000022215 | Fitm1 | -2.19 | 1.18E-02 |
| ENSMUSG00000052727 | Map1b | -2.20 | 0.00E+00 |
| ENSMUSG00000022324 | Matn2 | -2.22 | 2.82E-08 |
| ENSMUSG00000031661 | Nkd1 | -2.22 | 6.53E-06 |
| ENSMUSG00000022490 | Ppp1r1a | -2.23 | 1.70E-02 |
| ENSMUSG00000022548 | Apod | -2.23 | 2.29E-06 |
| ENSMUSG00000035105 | Egln3 | -2.24 | 3.33E-03 |
| ENSMUSG00000075307 | Klhl41 | -2.24 | 7.82E-04 |
| ENSMUSG00000002831 | Plin4 | -2.25 | 5.00E-02 |
| ENSMUSG00000070867 | Trabd2b | -2.25 | 3.27E-03 |
| ENSMUSG00000055653 | Gpc3 | -2.26 | 1.52E-06 |
| ENSMUSG00000039476 | Prrx2 | -2.27 | 0.00E+00 |
| ENSMUSG00000052374 | Actn2 | -2.27 | 8.63E-03 |
| ENSMUSG00000023092 | Fhl1 | -2.29 | 9.82E-07 |
| ENSMUSG00000071984 | Fndc1 | -2.29 | 1.24E-08 |
| ENSMUSG00000029348 | Asphd2 | -2.31 | 3.93E-06 |
| ENSMUSG00000041476 | Smpx | -2.32 | 5.05E-03 |
| ENSMUSG00000074218 | Cox7a1 | -2.32 | 6.20E-03 |
| ENSMUSG00000030862 | Cpxm2 | -2.33 | 6.53E-06 |
| ENSMUSG00000031561 | Tenm3 | -2.34 | 9.82E-07 |
| ENSMUSG00000038777 | Sema6c | -2.34 | 5.24E-05 |
| ENSMUSG00000047419 | Cmya5 | -2.34 | 1.80E-04 |
| ENSMUSG00000057751 | Megf6 | -2.36 | 1.19E-07 |
| ENSMUSG00000006221 | Hspb7 | -2.37 | 1.83E-04 |
| ENSMUSG00000056174 | Col8a2 | -2.37 | 2.00E-10 |
| ENSMUSG00000058297 | Spock2 | -2.37 | 1.94E-07 |
| ENSMUSG00000073889 | Il11ra1 | -2.39 | 0.00E+00 |
| ENSMUSG00000002500 | Rpl3l | -2.42 | 3.26E-03 |
| ENSMUSG00000025537 | Phkg1 | -2.44 | 1.14E-02 |
| ENSMUSG00000026489 | Coq8a | -2.44 | 5.76E-04 |
| ENSMUSG00000052316 | Lrrc15 | -2.45 | 0.00E+00 |
| ENSMUSG00000078486 | Perm1 | -2.45 | 1.01E-02 |
| ENSMUSG00000026043 | Col3a1 | -2.46 | 0.00E+00 |
| ENSMUSG00000048450 | Msx1 | -2.51 | 0.00E+00 |
| ENSMUSG00000096146 | Kcnj11 | -2.51 | 2.31E-02 |
| ENSMUSG00000040164 | Kcns1 | -2.54 | 8.31E-04 |
| ENSMUSG00000048616 | Nog | -2.54 | 9.25E-05 |
| ENSMUSG00000032925 | Itgbl1 | -2.55 | 1.13E-06 |
| ENSMUSG00000056328 | Myh1 | -2.55 | 6.17E-04 |
| ENSMUSG00000036334 | Igsf10 | -2.57 | 0.00E+00 |
| ENSMUSG00000019933 | Mrln | -2.58 | 1.70E-02 |
| ENSMUSG00000035606 | Ky | -2.60 | 8.15E-03 |
| ENSMUSG00000069171 | Nr2f1 | -2.62 | 1.00E-10 |
| ENSMUSG00000063415 | Cyp26b1 | -2.63 | 2.18E-07 |
| ENSMUSG00000025129 | Ppp1r27 | -2.64 | 7.85E-04 |
| ENSMUSG00000043795 | Prr33 | -2.65 | 9.46E-03 |
| ENSMUSG00000024471 | Myot | -2.66 | 2.33E-03 |
| ENSMUSG00000031448 | Adprhl1 | -2.67 | 1.55E-02 |
| ENSMUSG00000039405 | Prss23 | -2.68 | 0.00E+00 |
| ENSMUSG00000049641 | Vgll2 | -2.68 | 2.30E-03 |
| ENSMUSG00000016995 | Matn4 | -2.69 | 1.94E-07 |
| ENSMUSG00000036854 | Hspb6 | -2.71 | 2.14E-05 |
| ENSMUSG00000048583 | Igf2 | -2.72 | 4.16E-04 |
| ENSMUSG00000051048 | P4ha3 | -2.74 | 0.00E+00 |
| ENSMUSG00000021622 | Ckmt2 | -2.77 | 5.33E-04 |
| ENSMUSG00000028600 | Podn | -2.84 | 2.51E-04 |
| ENSMUSG00000020122 | Egfr | -2.85 | 1.85E-05 |
| ENSMUSG00000044951 | Mylk4 | -2.86 | 1.88E-02 |
| ENSMUSG00000031070 | Mrgprf | -2.88 | 5.45E-05 |
| ENSMUSG00000026725 | Tnn | -2.97 | 0.00E+00 |
| ENSMUSG00000017344 | Vtn | -3.04 | 1.23E-04 |
| ENSMUSG00000042436 | Mfap4 | -3.06 | 0.00E+00 |
| ENSMUSG00000030433 | Sbk2 | -3.08 | 8.04E-04 |
| ENSMUSG00000033544 | Angptl1 | -3.13 | 2.68E-05 |
| ENSMUSG00000070576 | Mn1 | -3.21 | 1.20E-07 |
| ENSMUSG00000015090 | Ptgds | -3.24 | 4.08E-03 |
| ENSMUSG00000018893 | Mb | -3.24 | 1.19E-07 |
| ENSMUSG00000031825 | Crispld2 | -3.25 | 0.00E+00 |
| ENSMUSG00000050010 | Shisa3 | -3.25 | 7.54E-04 |
| ENSMUSG00000030470 | Csrp3 | -3.29 | 1.78E-06 |
| ENSMUSG00000063821 | Dupd1 | -3.29 | 9.24E-04 |
| ENSMUSG00000033196 | Myh2 | -3.32 | 6.08E-06 |
| ENSMUSG00000027077 | Smtnl1 | -3.35 | 1.02E-06 |
| ENSMUSG00000037139 | Myom3 | -3.46 | 9.86E-07 |
| ENSMUSG00000027224 | Duoxa1 | -3.54 | 1.50E-06 |
| ENSMUSG00000063594 | Gng8 | -3.69 | 1.70E-03 |
| ENSMUSG00000046491 | C1qtnf2 | -3.92 | 0.00E+00 |
| ENSMUSG00000024134 | Six2 | -3.99 | 0.00E+00 |
| ENSMUSG00000040310 | Alx4 | -4.01 | 0.00E+00 |
| ENSMUSG00000027996 | Sfrp2 | -4.26 | 0.00E+00 |
| ENSMUSG00000029683 | Lmod2 | -4.43 | 2.68E-04 |
| ENSMUSG00000055775 | Myh8 | -4.55 | 0.00E+00 |
| ENSMUSG00000033268 | Duox1 | -4.71 | 6.02E-05 |
| ENSMUSG00000058665 | En1 | -4.77 | 1.17E-06 |
| ENSMUSG00000026574 | Dpt | -5.02 | 0.00E+00 |
| ENSMUSG00000036972 | Zic4 | -5.04 | 7.84E-07 |
| ENSMUSG00000048251 | Bcl11b | -5.22 | 4.91E-08 |
| ENSMUSG00000032368 | Zic1 | -6.25 | 1.00E-10 |

**Table S4:** DEGs found in mature animals by Wang et al. (2020) (15) and Rawlinson et al. (2009) (16) and expressed in juvenile mice (bold = differentially expressed in juvenile mouse too; *= max transcripts <15)

| Gene ID | Gene name | log2FC (Cortical/Calvarial) | p.adj | Gene ID | Gene name | log2FC (Cortical/Calvarial) | p.adj |
| --- | --- | --- | --- | --- | --- | --- | --- |
| **ENSMUSG00000036139** | **Hoxc9** | **4.89** | **4.30E-07** | ENSMUSG00000028717 | Tal1 | -0.01 | 9.99E-01 |
| ENSMUSG00000022484 | Hoxc10* | 4.75 | 2.97E-06 | ENSMUSG00000024868 | Dkk1 | -0.03 | 9.85E-01 |
| ENSMUSG00000075394 | Hoxc4* | 4.63 | 1.68E-05 | ENSMUSG00000020167 | Tcf3 | -0.04 | 9.43E-01 |
| **ENSMUSG00000001661** | **Hoxc6** | **4.11** | **4.95E-07** | ENSMUSG00000054191 | Klf1 | -0.05 | 9.67E-01 |
| **ENSMUSG00000000938** | **Hoxa10** | **3.93** | **1.12E-06** | ENSMUSG00000000782 | Tcf7 | -0.05 | 9.65E-01 |
| **ENSMUSG00000027833** | **Shox2** | **3.17** | **0.00E+00** | ENSMUSG00000030083 | Abtb1 | -0.07 | 9.07E-01 |
| ENSMUSG00000057777 | Mab21l2* | 3.04 | 1.11E-02 | ENSMUSG00000030894 | Tpp1 | -0.09 | 8.79E-01 |
| ENSMUSG00000038210 | Hoxa11* | 2.99 | 2.76E-02 | ENSMUSG00000055435 | Maf | -0.09 | 9.50E-01 |
| **ENSMUSG00000038236** | **Hoxa7** | **2.85** | **5.19E-08** | ENSMUSG00000051510 | Mafg | -0.11 | 8.25E-01 |
| ENSMUSG00000038253 | Hoxa5* | 2.61 | 1.55E-02 | ENSMUSG00000024140 | Epas1 | -0.11 | 7.99E-01 |
| **ENSMUSG00000027004** | **Frzb** | **1.98** | **1.67E-02** | ENSMUSG00000025151 | Maged1 | -0.11 | 8.58E-01 |
| ENSMUSG00000024924 | Vldlr | 1.40 | 1.45E-01 | ENSMUSG00000025330 | Padi4 | -0.12 | 9.11E-01 |
| ENSMUSG00000029671 | Wnt16 | 1.19 | 3.59E-01 | ENSMUSG00000046179 | E2f8 | -0.12 | 9.17E-01 |
| ***ENSMUSG00000031962*** | ***Cdh15*** | ***0.86*** | ***5.00E-02*** | ENSMUSG00000062906 | Hdac10 | -0.13 | 8.55E-01 |
| ENSMUSG00000038319 | Kcnh2 | 0.83 | 4.38E-01 | ENSMUSG00000026815 | Gfi1b | -0.13 | 8.76E-01 |
| ENSMUSG00000027177 | Hipk3 | 0.63 | 3.14E-01 | ENSMUSG00000037235 | Mxd4 | -0.14 | 7.14E-01 |
| ENSMUSG00000038331 | Satb2 | 0.63 | 1.10E-01 | ENSMUSG00000026678 | Rgs5 | -0.14 | 9.30E-01 |
| ***ENSMUSG00000001348*** | ***Acp5*** | ***0.62*** | ***4.09E-02*** | ENSMUSG00000014599 | Csf1 | -0.14 | 7.79E-01 |
| ENSMUSG00000029306 | Ibsp | 0.57 | 3.47E-01 | ENSMUSG00000021250 | Fos | -0.15 | 9.03E-01 |
| ENSMUSG00000036943 | Rab8b | 0.54 | 7.24E-01 | ENSMUSG00000070436 | Serpinh1 | -0.15 | 8.29E-01 |
| ENSMUSG00000029335 | Bmp3 | 0.50 | 8.22E-01 | ENSMUSG00000025584 | Pde8a | -0.16 | 8.39E-01 |
| ENSMUSG00000052435 | Cebpe | 0.45 | 5.78E-01 | ENSMUSG00000001131 | Timp1 | -0.17 | 8.44E-01 |
| ENSMUSG00000017737 | Mmp9 | 0.44 | 3.41E-01 | ENSMUSG00000040097 | Flywch1 | -0.18 | 7.69E-01 |
| ENSMUSG00000031162 | Gata1 | 0.43 | 4.90E-01 | ENSMUSG00000070803 | Cited4 | -0.18 | 8.48E-01 |
| ENSMUSG00000018604 | Tbx3 | 0.42 | 8.43E-01 | ENSMUSG00000031565 | Fgfr1 | -0.19 | 6.90E-01 |
| ENSMUSG00000031849 | Comp | 0.42 | 7.60E-01 | ENSMUSG00000024803 | Ankrd1 | -0.20 | 9.44E-01 |
| ENSMUSG00000054196 | Cthrc1 | 0.38 | 5.15E-01 | ENSMUSG00000027230 | Creb3l1 | -0.20 | 7.73E-01 |
| ENSMUSG00000028111 | Ctsk | 0.37 | 2.91E-01 | ENSMUSG00000038151 | Prdm1 | -0.20 | 9.12E-01 |
| ENSMUSG00000032179 | Bmp5 | 0.33 | 7.30E-01 | ENSMUSG00000026028 | Trak2 | -0.21 | 7.18E-01 |
| ENSMUSG00000050578 | Mmp13 | 0.31 | 8.29E-01 | ENSMUSG00000029781 | Fkbp9 | -0.22 | 6.51E-01 |
| ENSMUSG00000024621 | Csf1r | 0.29 | 3.97E-01 | ENSMUSG00000025902 | Sox17 | -0.23 | 8.32E-01 |
| ENSMUSG00000036585 | Fgf1 | 0.25 | 7.88E-01 | ENSMUSG00000021493 | Pdlim7 | -0.23 | 6.67E-01 |
| ENSMUSG00000017421 | Zfp207 | 0.25 | 4.04E-01 | ENSMUSG00000022528 | Hes1 | -0.25 | 6.21E-01 |
| ENSMUSG00000060284 | Sp7 | 0.25 | 6.91E-01 | ENSMUSG00000063870 | Chd4 | -0.26 | 1.28E-01 |
| ENSMUSG00000022905 | Kpna1 | 0.23 | 8.67E-01 | ENSMUSG00000053477 | Tcf4 | -0.26 | 3.32E-01 |
| ENSMUSG00000063727 | Tnfrsf11b | 0.23 | 8.64E-01 | ENSMUSG00000022246 | Rai14 | -0.27 | 7.19E-01 |
| ENSMUSG00000027560 | Dok5 | 0.23 | 8.55E-01 | ENSMUSG00000035112 | Wnk4 | -0.27 | 8.36E-01 |
| ENSMUSG00000026311 | Asb1 | 0.18 | 8.34E-01 | ENSMUSG00000027868 | Tbx15 | -0.29 | 7.19E-01 |
| ENSMUSG00000029755 | Dlx5 | 0.16 | 8.54E-01 | ENSMUSG00000022479 | Vdr | -0.29 | 5.01E-01 |
| ENSMUSG00000036596 | Cpz | 0.15 | 8.43E-01 | ENSMUSG00000003545 | Fosb | -0.31 | 9.10E-01 |
| ENSMUSG00000001510 | Dlx3 | 0.14 | 8.88E-01 | ENSMUSG00000018143 | Mafk | -0.31 | 5.89E-01 |
| ENSMUSG00000000093 | Tbx2 | 0.13 | 8.64E-01 | ENSMUSG00000030551 | Nr2f2 | -0.32 | 7.12E-01 |
| ENSMUSG00000005800 | Mmp8 | 0.13 | 9.56E-01 | ENSMUSG00000041842 | Fhdc1 | -0.34 | 5.62E-01 |
| ENSMUSG00000058794 | Nfe2 | 0.13 | 8.98E-01 | ENSMUSG00000074766 | Ism1 | -0.35 | 7.09E-01 |
| ENSMUSG00000006932 | Ctnnb1 | 0.13 | 7.91E-01 | ENSMUSG00000062590 | Armc9 | -0.35 | 6.29E-01 |
| ENSMUSG00000020423 | Btg2 | 0.08 | 9.38E-01 | ENSMUSG00000035686 | Thrsp | -0.36 | 8.12E-01 |
| ENSMUSG00000003585 | Sec14l2 | 0.04 | 9.69E-01 | ENSMUSG00000051910 | Sox6 | -0.43 | 6.01E-01 |
| ENSMUSG00000003355 | Fkbp11 | 0.04 | 9.66E-01 | ENSMUSG00000029309 | Sparcl1 | -0.43 | 3.41E-01 |
| ENSMUSG00000022952 | Runx1 | 0.01 | 9.82E-01 | ENSMUSG00000035914 | Cd276 | -0.44 | 3.88E-01 |
|  |  |  |  | ENSMUSG00000029922 | Mkrn1 | -0.47 | 3.19E-01 |
|  |  |  |  | ENSMUSG00000020218 | Wif1 | -0.50 | 2.58E-02 |
|  |  |  |  | ENSMUSG00000078566 | Bnip3 | -0.54 | 6.33E-01 |
|  |  |  |  | ENSMUSG00000020303 | Stc2 | -0.57 | 8.26E-01 |
|  |  |  |  | ENSMUSG00000024913 | Lrp5 | -0.58 | 7.84E-02 |
|  |  |  |  | ENSMUSG00000033581 | Igf2bp2 | -0.59 | 5.07E-01 |
|  |  |  |  | ENSMUSG00000073557 | Ppp1r12b | -0.60 | 1.38E-01 |
|  |  |  |  | ENSMUSG00000025402 | Nab2 | -0.61 | 1.49E-01 |
|  |  |  |  | ENSMUSG00000024236 | Svil | -0.61 | 6.72E-02 |
|  |  |  |  | ENSMUSG00000030208 | Emp1 | -0.63 | 1.01E-01 |
|  |  |  |  | ENSMUSG00000037370 | Enpp1 | -0.64 | 8.19E-02 |
|  |  |  |  | **ENSMUSG00000008575** | **Nfib** | **-0.65** | **3.06E-02** |
|  |  |  |  | ENSMUSG00000041577 | Prelp | -0.78 | 3.85E-01 |
|  |  |  |  | **ENSMUSG00000027253** | **Lrp4** | **-0.82** | **1.12E-02** |
|  |  |  |  | ENSMUSG00000001494 | Sost | -0.92 | 6.86E-02 |
|  |  |  |  | **ENSMUSG00000022265** | **Ank** | **-0.94** | **8.85E-08** |
|  |  |  |  | **ENSMUSG00000029675** | **Eln** | **-0.99** | **1.04E-02** |
|  |  |  |  | **ENSMUSG00000026185** | **Igfbp5** | **-1.09** | **5.00E-10** |
|  |  |  |  | ***ENSMUSG00000053863*** | ***Mepe*** | ***-1.15*** | ***4.20E-04*** |
|  |  |  |  | ENSMUSG00000025255 | Zfhx4 | -1.18 | 1.71E-01 |
|  |  |  |  | ENSMUSG00000027210 | Meis2 | -1.33 | 2.23E-01 |
|  |  |  |  | ENSMUSG00000026407 | Cacna1s | -1.57 | 8.94E-02 |
|  |  |  |  | **ENSMUSG00000023411** | **Nfatc4** | **-1.60** | **5.33E-05** |
|  |  |  |  | **ENSMUSG00000028664** | **Ephb2** | **-1.66** | **7.67E-04** |
|  |  |  |  | **ENSMUSG00000035799** | **Twist1** | **-1.69** | **1.00E-10** |
|  |  |  |  | **ENSMUSG00000029838** | **Ptn** | **-1.73** | **5.20E-09** |
|  |  |  |  | **ENSMUSG00000038872** | **Zfhx3** | **-1.88** | **1.40E-05** |
|  |  |  |  | **ENSMUSG00000097993** | **Ptprv** | **-2.09** | **1.75E-06** |
|  |  |  |  | ENSMUSG00000055027 | Smyd1 | -2.35 | 8.83E-02 |
|  |  |  |  | **ENSMUSG00000048450** | **Msx1** | **-2.51** | **0.00E+00** |
|  |  |  |  | **ENSMUSG00000048616** | **Nog** | **-2.54** | **9.25E-05** |
|  |  |  |  | **ENSMUSG00000069171** | **Nr2f1** | **-2.62** | **1.00E-10** |
|  |  |  |  | **ENSMUSG00000063415** | **Cyp26b1** | **-2.63** | **2.18E-07** |
|  |  |  |  | ***ENSMUSG00000030470*** | ***Csrp3*** | ***-3.29*** | ***1.78E-06*** |
|  |  |  |  | **ENSMUSG00000027996** | **Sfrp2** | **-4.26** | **0.00E+00** |
|  |  |  |  | **ENSMUSG00000036972** | **Zic4** | **-5.04** | **7.84E-07** |
|  |  |  |  | **ENSMUSG00000032368** | **Zic1** | **-6.25** | **1.00E-10** |

**Table S5:** GSEA using KEGG pathway gene sets

| Gene Set ID | Description | setSize | enrichmentScore | NES | p.value | p.adj | qvalue | core enrichment |
| --- | --- | --- | --- | --- | --- | --- | --- | --- |
| mmu03010 | Ribosome | 129 | 0.66 | 3.12 | 1.00E-10 | 1.69E-08 | 1.43E-08 | Rps17 Rpl4 Mrpl20 Rpl36al Rpl35 Rpl11 Rps3 Rpl31 Rpl32 Rpl34 Rpl9 Rpl23 Rps27l Rpl27a Rps20 Rpl30 Rpl12 Rpl22 Rpl6 Rps7 Mrpl15 Rpl26 Rpl18 Rpl41 Rps3a1 Rpl36a Rpl23a Rpl10 Rpl7 Rps29 Rpl10a Rpl3 Rps13 Mrpl11 Rpl37 Rpl32l Rpl15 Rpl29 Rps18 Rps11 Rpl5 Rps21 Rps2 Mrpl18 Mrpl30 Mrpl24 Rpl27 Mrpl27 Rps27a Mrpl21 Rpl14 Rpl35a Rps5 Rpl17 Rps6 Rpl36 Mrps16 Mrpl36 Rpl13a Rps23 Mrps15 Fau Rpsa Rps10 Mrps14 Rplp0 Mrps21 Rpl21 Rpl18a Mrps2 Mrps18c Rpl13 Rps15 Rpl8 Rps19 Mrpl28 Rps26 Rpl39l Mrpl13 Rpl28 Rpl7a Mrps7 Rsl24d1 Mrps17 Rpl37rt Rps14 Mrpl22 |
| mmu05171 | Coronavirus disease - COVID-19 | 163 | 0.51 | 2.48 | 1.00E-10 | 1.69E-08 | 1.43E-08 | Rps17 Rpl4 Rpl36al Rpl35 Rpl11 Rps3 Rpl31 Rpl32 Rpl34 Rpl9 Rpl23 Rps27l Rpl27a Rps20 Rpl30 Rpl12 Rpl22 Rpl6 Rps7 Rpl26 Rpl18 Rpl41 Rps3a1 Rpl36a Rpl23a F13a1 Rpl10 Rpl7 Rps29 Rpl10a Rpl3 Rps13 Rpl37 Cxcl10 Rpl32l Rpl15 Rpl29 Rps18 Rps11 Rpl5 Rps21 Rps2 Rpl27 Rps27a Rpl14 Rpl35a Cybb Rps5 Rpl17 Rps6 Myd88 Rpl36 Rpl13a Rps23 Fau Rpsa Rps10 Rplp0 C3 Rpl21 Rpl18a Rpl13 Rps15 Rpl8 Mapk13 Rps19 Stat1 Pik3cd Prkca Jak1 Ifnar2 Rps26 Rpl39l C4b Rpl28 Rpl7a Tlr2 Rsl24d1 Eif2ak2 Rpl37rt Rps14 |
| mmu05414 | Dilated cardiomyopathy | 61 | -0.61 | -2.21 | 3.40E-07 | 3.83E-05 | 3.24E-05 | Adcy4 Sgca Itga7 Tpm1 Adcy2 Adcy6 Cacna1s Prkaca Cacnb1 Cacna1c Des Ttn Tpm2 Itga11 Cacng6 Atp2a1 Itgb4 Cacng1 Lama2 Tgfb2 Dag1 Lmna Tgfb3 Itgb5 |
| mmu03050 | Proteasome | 41 | 0.62 | 2.38 | 7.85E-07 | 6.63E-05 | 5.60E-05 | Psmb1 Psmb3 Psma6 Pomp Psmb6 Psmb4 Psma7 Psme1 Psma5 Psme2 Psmd6 Psmb8 Psmd11 Psmb7 Psmb9 Psmc4 Psma2 Psmd12 Psmd14 Psmc2 Psmc1 Psmd8 |
| mmu03030 | DNA replication | 34 | 0.65 | 2.34 | 1.00E-06 | 6.78E-05 | 5.72E-05 | Ssbp1 Rpa3 Rfc3 Pole4 Rfc4 Pola1 Rnaseh2b Pole Rnaseh2c Mcm6 Rfc2 Pcna Rfc1 Prim2 Mcm5 Rfc5 Pold3 Rpa2 |
| mmu03040 | Spliceosome | 116 | 0.44 | 2.07 | 1.35E-06 | 7.58E-05 | 6.40E-05 | Snu13 Lsm6 Eif4a3 Snrpd3 Bud31 Snrpg Hnrnpc Hnrnpu Snrpd1 Dhx15 Cwc15 Alyref Srsf3 Snrpb Ddx46 Snrpe Srsf7 Lsm8 Hnrnpk Snrpf Phf5a Puf60 Thoc3 Snrpa1 Srsf2 Lsm7 U2af1 Hnrnpa1 Srsf9 Rbm17 Ppil1 Ctnnbl1 Cdc5l Bcas2 Srsf1 Pcbp1 Sf3a3 Hnrnpm Ddx5 Ncbp1 Zmat2 Prpf40a Eftud2 Hspa8 Txnl4a Aqr Crnkl1 Magohb Sf3b5 Usp39 Sf3b3 Snrnp40 Prpf38a Rbm8a Srsf10 Snrpd2 Tra2b Cdc40 Pqbp1 Prpf31 Lsm4 |
| mmu05410 | Hypertrophic cardiomyopathy | 57 | -0.59 | -2.14 | 3.55E-06 | 1.71E-04 | 1.45E-04 | Prkab2 Itga3 Sgca Itga7 Tpm1 Ace Cacna1s Cacnb1 Cacna1c Des Ttn Tpm2 Itga11 Cacng6 Atp2a1 Itgb4 Cacng1 Lama2 Tgfb2 Dag1 Lmna Tgfb3 Itgb5 |
| mmu04340 | Hedgehog signaling pathway | 40 | -0.63 | -2.11 | 1.16E-05 | 4.90E-04 | 4.13E-04 | Ptch1 Sufu Ccnd1 Mgrn1 Megf8 Csnk1e Ccnd2 Kif3a Spop Csnk1d Csnk1g2 Smurf1 Iqce Gas1 Disp1 Smo Hhatl Prkaca Evc Boc Evc2 Scube2 Gli1 |
| mmu04613 | Neutrophil extracellular trap formation | 107 | 0.42 | 1.95 | 1.71E-05 | 6.42E-04 | 5.42E-04 | Ctsg Slc25a5 Mpo Itgb3 Elane H2az1 Hdac2 H3c13 H2ac22 Plcb2 Cyba Vdac2 Macroh2a1 Ncf2 H3c3 Cybb Ncf1 H3f3a Camp H3f3b H2ac13 H2ac8 Itgb2 H2bc11 Selplg H2ac11 Itgb2l H2aj C3 Rac1 Mapk13 Pik3cd Prkca H2ac7 H2ac20 H2ac4 Tlr2 Ncf4 H2ac24 H3c1 H2ac15 Map3k7 Plcb3 H4c9 Itga2b H4c1 Vdac3 |
| mmu05412 | Arrhythmogenic right ventricular cardiomyopathy | 47 | -0.59 | -2.04 | 3.22E-05 | 1.09E-03 | 9.20E-04 | Sgca Itga7 Cacna1s Cacnb1 Cacna1c Des Itga11 Tcf7l1 Cacng6 Atp2a1 Itgb4 Actn2 Cacng1 Lama2 Dag1 Lmna Itgb5 |
| mmu04010 | MAPK signaling pathway | 183 | -0.40 | -1.72 | 7.61E-05 | 2.34E-03 | 1.97E-03 | Dusp7 Nfkb2 Gadd45b Erbb2 Dusp1 Flna Dusp8 Gna12 Taok3 Rela Flt1 Mapk8ip3 Map2k7 Araf Elk1 Rps6ka2 Nf1 Map3k20 Mras Srf Gadd45g Hspa1a Fgfr3 Mapk8ip1 Flnb Rras Map2k2 Map3k6 Tab1 Hspa1b Relb Efna5 Ntrk2 Cacna1s Prkaca Map3k11 Cacnb1 Jun Cacna1c Nr4a1 Cacng6 Il1r1 Efna2 Map2k3 Jund Cacng1 Vegfb Tgfb2 Pdgfrb Hspb1 Igf2 Flnc Tgfb3 Cacna1g |
| mmu03060 | Protein export | 20 | 0.67 | 2.14 | 8.81E-05 | 2.43E-03 | 2.05E-03 | Srp9 Srp19 Sec61b Spcs2 Srp14 Spcs1 Spcs3 Sec11c Srp72 Hspa5 |
| mmu04512 | ECM-receptor interaction | 64 | -0.53 | -1.96 | 9.87E-05 | 2.43E-03 | 2.05E-03 | Col4a2 Itga3 Col4a1 Hspg2 Itga7 Lama4 Lamc1 Tnxb Vwf Lamb1 Itga11 Lamb2 Itgb4 Lama2 Dag1 Thbs3 Npnt Vtn Col6a3 Col6a2 Thbs2 Col6a1 Tnn Itgb5 |
| mmu03430 | Mismatch repair | 21 | 0.67 | 2.14 | 1.00E-04 | 2.43E-03 | 2.05E-03 | Ssbp1 Rpa3 Rfc3 Rfc4 Rfc2 Pcna Rfc1 Rfc5 Pold3 Rpa2 |
| mmu04510 | Focal adhesion | 156 | -0.42 | -1.76 | 1.45E-04 | 3.26E-03 | 2.76E-03 | Myl12a Ccnd1 Thbs4 Erbb2 Ppp1r12c Flna Ccnd2 Mylk2 Flt1 Col4a2 Pak4 Rapgef1 Itga3 Pik3r2 Col4a1 Elk1 Mylpf Itga7 Lama4 Flnb Actn1 Ppp1r12b Lamc1 Tnxb Parva Tln2 Vwf Lamb1 Jun Myl9 Itga11 Mylk4 Lamb2 Actn4 Itgb4 Lama2 Vegfb Cav3 Pdgfrb Thbs3 Vtn Flnc Col6a3 Col6a2 Thbs2 Mylk Col6a1 Tnn Itgb5 |
| mmu03440 | Homologous recombination | 28 | 0.59 | 2.01 | 3.39E-04 | 7.16E-03 | 6.05E-03 | Ssbp1 Rpa3 Eme1 Rbbp8 Pold3 Rad52 Rpa2 Rad54b Blm Mre11a Babam1 Nbn Top3b Babam2 Pold4 Rad50 Rpa1 |
| mmu03420 | Nucleotide excision repair | 37 | 0.54 | 2.00 | 3.92E-04 | 7.80E-03 | 6.58E-03 | Rpa3 Gtf2h5 Rfc3 Pole4 Rfc4 Ccnh Pole Rfc2 Pcna Xpc Rfc1 Rfc5 Pold3 Rpa2 Gtf2h1 Rbx1 |
| mmu04020 | Calcium signaling pathway | 109 | -0.43 | -1.71 | 7.69E-04 | 1.44E-02 | 1.22E-02 | Adcy4 Sphk1 Itpr2 Casq1 Fgfr3 Adcy2 Plcd3 Gna11 Ntrk2 Cacna1s Prkaca Ryr1 Tpcn1 Cacna1c Tnnc2 Ednra Hrc Camk2g Asph Mylk4 Atp2a1 Phkg1 Vegfb Camk2a Slc25a4 Pdgfrb Stim1 Mylk Cacna1g |
| mmu00983 | Drug metabolism - other enzymes | 34 | 0.51 | 1.86 | 1.14E-03 | 1.95E-02 | 1.65E-02 | Mpo Nme1 Upp1 Itpa Dut Uck2 Mgst1 Gsto1 Gstm1 Mgst2 Gstp3 Hprt Uckl1 Impdh1 Rrm1 Nme6 Nme4 Gstm5 Rrm2 Gstp1 Gusb Tk1 Ces2g Impdh2 |
| mmu04310 | Wnt signaling pathway | 106 | -0.41 | -1.66 | 1.16E-03 | 1.95E-02 | 1.65E-02 | Tle2 Tle3 Rock2 Ppp3cb Notum Axin1 Ryk Camk2b Ctbp2 Porcn Ccnd1 Plcb4 Wnt7b Tle1 Daam2 Csnk1e Ppard Ccnd2 Ctnnbip1 Dvl2 Serpinf1 Gpc4 Wnt5b Smad4 Smad3 Prkaca Lrp5 Sost Jun Lrp6 Dvl1 Wif1 Axin2 Tcf7l1 Camk2g Nfatc2 Camk2a Nfatc4 Ror2 Fzd1 Sfrp2 |
| mmu03008 | Ribosome biogenesis in eukaryotes | 55 | 0.43 | 1.75 | 2.09E-03 | 3.33E-02 | 2.81E-02 | Nhp2 Snu13 Rexo2 Pop4 Nop58 Csnk2b Utp4 Emg1 Heatr1 Eif6 Riok1 Nvl Lsg1 Dkc1 Nop56 Wdr3 Xrn2 Nxt1 Utp14a Nob1 |
| mmu05146 | Amoebiasis | 51 | -0.50 | -1.75 | 2.17E-03 | 3.33E-02 | 2.81E-02 | Rela Col4a2 Pik3r2 Col4a1 Lama4 Actn1 Gna11 Lamc1 Prkaca Lamb1 Il1r1 Lamb2 Actn4 Lama2 Rab7b Tgfb2 Hspb1 Tgfb3 Col3a1 |
| mmu01232 | Nucleotide metabolism | 59 | 0.42 | 1.73 | 3.40E-03 | 4.84E-02 | 4.09E-02 | Entpd3 Ada Nme1 Tyms Upp1 Itpa Dut Uck2 Hddc2 Ak2 Pnp Dtymk Ctps Nt5c Dctpp1 Hprt Pnp2 Cmpk2 Uckl1 Adss Adk Impdh1 Rrm1 Ntpcr |
| mmu04260 | Cardiac muscle contraction | 57 | -0.47 | -1.70 | 3.44E-03 | 4.84E-02 | 4.09E-02 | Tpm1 Uqcrc1 Atp1b2 Cox7c Cacna1s Cacnb1 Cox8b Cacna1c Tpm2 Hrc Asph Cacng6 Atp1a2 Atp2a1 Cox6a2 Slc9a1 Cox7a1 Cacng1 |
| mmu04270 | Vascular smooth muscle contraction | 78 | -0.43 | -1.63 | 3.74E-03 | 4.92E-02 | 4.15E-02 | Myh9 Myh14 Itpr3 Pla2g5 Plcb4 Ppp1r12c Gna12 Prkce Mylk2 Araf Myh10 Adcy4 Itpr2 Adcy2 Cald1 Map2k2 Arhgef11 Ppp1r12b Npr2 Gna11 Adcy6 Ramp2 Cacna1s Prkaca Kcnmb4 Myh11 Myl9 Cacna1c Ednra Mylk4 Acta2 Mylk |
| mmu05322 | Systemic lupus erythematosus | 54 | 0.41 | 1.66 | 3.78E-03 | 4.92E-02 | 4.15E-02 | Ctsg Elane Snrpd3 H2az1 H3c13 H2ac22 Snrpd1 Macroh2a1 Snrpb H3c3 H3f3a H3f3b H2-Ob H2ac13 H2ac8 H2bc11 H2ac11 Cd40 H2aj C3 H2ac7 H2ac20 C4b H2ac4 H2ac24 H2-DMa H3c1 H2ac15 |
